# Polycarbazole and Its Derivatives: Synthesis and Applications. A Review of the Last 10 Years

**DOI:** 10.3390/polym12102227

**Published:** 2020-09-28

**Authors:** Fadila Bekkar, Faiza Bettahar, Isabel Moreno, Rachid Meghabar, Mohammed Hamadouche, Estibaliz Hernáez, José Luis Vilas-Vilela, Leire Ruiz-Rubio

**Affiliations:** 1 Laboratoire de Chimie des Polymères, Université Oran1 Ahmed Ben Bella, El-Mnaouer, BP 1524, Oran 31000, Algerie; bekkar31@outlook.com (F.B.); faiza-bettahar@hotmail.com (F.B.); rachidmeghabar@yahoo.fr (R.M.); 2 Macromolecular Chemistry Group (LQM), Organic Chemistry II Department, Faculty of Science and Technology, University of the Basque Country (UPV/EHU), 48940 Leioa, Spain; mariaisabel.moreno@ehu.eus; 3 Laboratoire de Chimie Fine, Département de Chimie, Faculté des Sciences Exactes et Appliquées, Université Oran1 Ahmed Ben Bella, El-Mnaouer, BP 1524, Oran 31000, Algerie; hamadouchemed@yahoo.fr; 4 Macromolecular Chemistry Group (LQM), Physical Chemistry Department, Faculty of Science and Technology, University of the Basque Country (UPV/EHU), 48940 Leioa, Spain; estibaliz.hernaez@ehu.eus (E.H.); joseluis.vilas@ehu.eus (J.L.V.-V.); 5 BCMaterials, Basque Center for Materials, Applications and Nanostructures, UPV/EHU Science Park, 48940 Leioa, Spain

**Keywords:** carbazole derivatives, conducting polymers, electropolymerization, chemical polymerization

## Abstract

Polycarbazole and its derivatives have been extensively used for the last three decades, although the interest in these materials briefly decreased. However, the increasing demand for conductive polymers for several applications such as light emitting diodes (OLEDs), capacitators or memory devices, among others, has renewed the interest in carbazole-based materials. In this review, the synthetic routes used for the development of carbazole-based polymers have been summarized, reviewing the main synthetic methodologies, namely chemical and electrochemical polymerization. In addition, the applications reported in the last decade for carbazole derivatives are analysed. The emergence of flexible and wearable electronic devices as a part of the internet of the things could be an important driving force to renew the interest on carbazole-based materials, being conductive polymers capable to respond adequately to requirement of these devices.

## 1. Introduction

Electrically conductive polymers are a highly demanded class of materials due to their extended uses in electronic and optical devices or sensors, among others. These materials present main advantageous properties of conventional polymers, such as solubility, mechanical flexibility, non-expensive fabrication or processing, with conductivity levels that could be compared to those of semiconductors or even metals. In 1977, Macdiarmid, Shirakawa and Heeger demonstrated that chemical doping of polyacetylene (PA) resulted in a highly conducting material, with its conductivity being eleven orders of magnitude higher than a pristine polymer. For this they were awarded with the Nobel Prize in 2000 for their research on conductive polymers [1,2]. These materials have induced the development of many applications, such as organic light emitting diodes (OLEDs) [3,4,5,6], organic field effect transistors (OFETs) [7,8,9,10,11], dye-sensitized solar cells [12,13,14,15,16], photochromic dyes [17,18,19,20,21], batteries [22,23,24,25,26], and electrocatalyst [27,28] or (bio)sensors [26,29,30,31,32]

**Figure 1:**
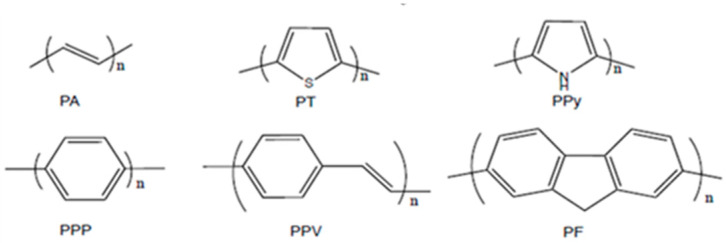
The structure of some of the main conjugated polymers.

The term conductive polymer encompasses both organic molecules containing of alternating simple (type σ) and double (type π) carbon bonds and/or conjugated aromatic nuclei on their skeleton. This particularity, π-conjugated structure, allows the transfer of charges (electrons or holes) along the macromolecular skeleton. The most commonly used conjugated polymers are poly(thiophene) (PT) [33,34], poly(pyrrole) (PPy) [35], poly(p-phenylene) (PPP) [36], poly (p-phenylenevinylene) (PPV) [37], and polyfluorene (PF) [38,39,40,41], with their structures summarized in Figure 1. In addition to the processability inherent of many polymers, it is important to notice their ability to modulate their conductivity by doping, which could vary from the isolation state (<10^−10^ S.cm^−1^), to the semiconductor state (~10^−5^ S.cm^−1^), and even to the conductive material (>10^4^ S.cm^−1^) near copper (5 × 10^5^ S.cm^−1^). Among electronic conductive polymers, polymers containing a carbazole ring (PCz) also present good electrical and optical properties.

**Figure 2:**
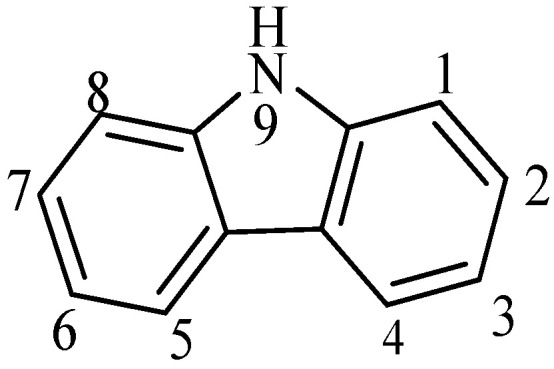
Structure of carbazole.

Carbazole (C_12_H_9_N), also named dibenzopyrrole or diphenylenimine, is an N-containig heterocyclic compound discovered by Graebe and Glaser in 1872 [42]. Its structure consists of two benzene ring fused on either side of a central pyrrole ring (Figure 2) [43]. Carbazole represents an important class of heterocycles with several advantages. For example, a large variety of substituents can be easily introduced in the nitrogen atom and the aromatic framework can be substituted in positions 3 and 6, modifying the physicochemical properties. Carbazol-based polymers (PCz) have attracted increased attention over the last 50 years owing to their stability and higher redox potential compared to other conducting polymers [44]. Similarly, they present good electro- and photoactive properties because of their high hole transporting mobility and strong absorption in the UV spectral region [45]. These characteristics have extended the use of this kind of polymer in several applications, such as transistors [46,47], smart windows [48], light emitting diode [49,50,51,52], (bio)sensor [53,54,55], and photovoltaic devices [56,57].

**Figure 3:**
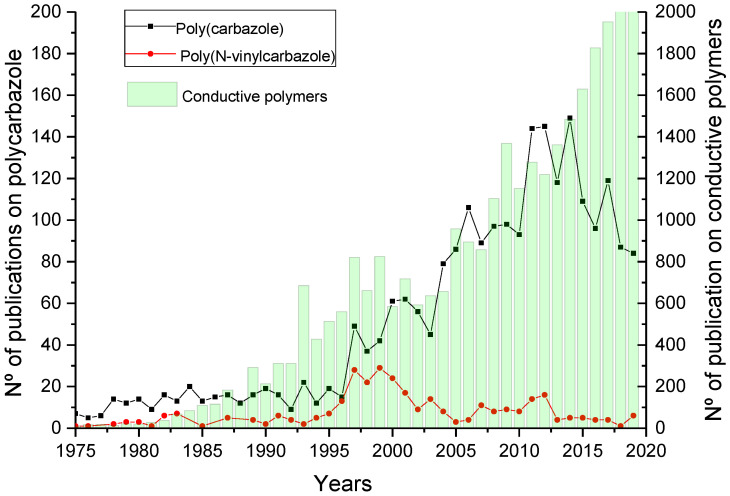
The evolution on the number of publications of conductive polymers (green), polycarbazole derivatives (black) and poly(N-vinylcarbazole) (red) (Source: Scopus).

The field of the conductive polymers has exponentially increased since 1985. The growth of the last decade has been favoured by the increase in devices related to the internet of things (IoT). These types of devices require new materials capable of satisfying their high requirements, with conductive polymers being one of the most widely used materials for these devices [57,58,59,60,61,62,63,64,65,66,67,68,69]. Among the conductive polymers, polycarbazole derivatives have also received increasing attention. As can be observed in Figure 3, there is a relation between the increase on the number of publications related to conductive polymers (green) and the increase of publications based on polycarbazole derivatives (red). However, this increase has been significantly lower for the case of poly(N-vinylcarbazole) (black).

In recent years, it is important to highlight the work of Mario Leclerc et al. with several reviews and book chapters focused on the synthesis of poly(3,6-carbazole) and poly(2,7-carbazole) derivatives for plastic electronics and solar cells [70,71,72,73,74]. Considering the Scopus database, in a total of 17 reviews (eight in the last 10 years) and rive chapters (four in the last 10 years) polycarbazole derivatives are mentioned. Table 1 and Table 2 summarize this information. However, in many of them, polycarbazole derivatives are part of the publication, but such publications are not completely devoted to them, or the publication is focused only on one type of derivative and/or application.

Considering the previous mentioned lacks, in this review, a global overview of the main polycarbazole derivatives, focused in the last decade, and their applications are revised. The main synthetic routes used for the development of carbazole-based polymers have been summarized in two parts: chemical and electrochemical polymerization of carbazole and its derivatives. In addition, the applications reported in the last years for carbazole-based polymers are reviewed (2010–2020).

## 2. Synthesis of Polymers

The synthesis and characterization of conducting polymers have become one of the most important areas of the research in polymer and materials science. In general, conductive polymers can be synthesized by different methods, with chemical polymerization (classical organic synthesis) and electrochemical polymerization (electrochemical synthesis) being the most commonly used processes. The following sections describe these two different synthesis techniques of polycarbazole and its derivatives.

Carbazole presents several active positions (3,6-, 2,7-, and 1,8-positions), being the 3,6 postions easier to polymerize. Poly(N-vinylcarbazole) and its derivatives has been highly studied since decades, but in the last decade 3,6-carbazole derivatives have been intensively investigated. Nevertheless, these derivatives present several limitations in their application due to their low molecular weight and poor conjugation of the electrons in their structure. On the other hand, the development of 2,7-carbazoles present better properties and applicability than the 3,6 due to their extended conjugation and lower band gap [70]. Finally, poly(1,8-carbazole) derivatives are the less developed derivatives, and poly(1,8-cabazole) is less planar compared to 3,6 and 2,7 [93,94]. However, this property makes these derivatives more suitable for the electrets of photoresponsive organic field-effect transistor memory applications [46].

### 2.1. Chemical Polymerization of Carbazoles and Its Derivatives

Chemical polymerization of carbazole has been carried out in the presence of oxidizing agents such as ammonium persulphate ((NH_4_)_2_S_2_O_8_) (APS), ferric chloride (FeCl_3_), and potassium dichromate (K_2_Cr_2_O_7_). The structure and properties of the obtained polymer are strongly dependent on the concentration, the catalyst (oxidizing agent), and the solvent [74]. The chemical synthesis takes place by oxidation-reduction reactions that are accompanied by a change in the number of electrons in the π system. The first studies on the oxidation of carbazole were published by Branch and Tucker [95,96,97]. Although the most commonly used dopants are FeCl_3_ or I_2_, these oxidants can promote polymer aggregation and, as consequence, important problems on the device production. To overcome this drawback Aoai et al. [98] described a photodoping method based on the use of triaylsulfonium or diaryliodonium salts as PAG (photo acid generator).

**Figure 4:**
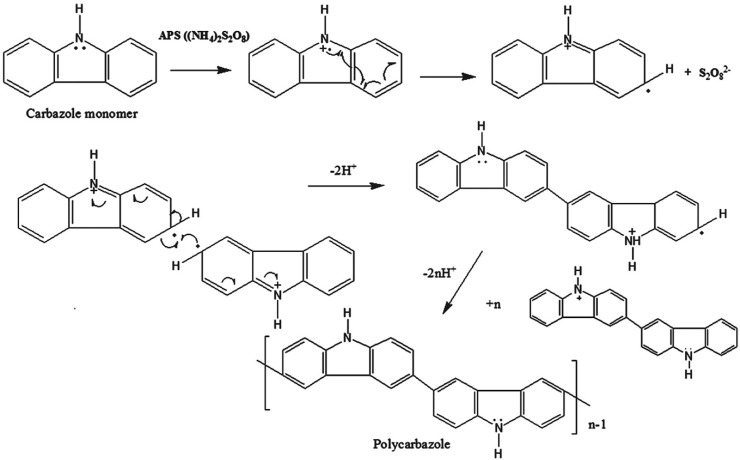
Proposed mechanistic scheme for the synthesis of carbazol-based polymers (PCz). Reprinted with permission from Sangwan et al. [99]. Copyright (2016) Wiley.

The mechanism proposed for the synthesis of PCz (or poly(3,6-cabazole)) is presented in Figure 4. As depicted in Figure 4, first, carbazole monomer is oxidized by a single electron transfer forming the cation radical. Then more stable dicarbazyl dimer is produced as a result of the coupling of two cation radicals and the loss of two protons. Regarding the regiochemistry, the polymerization process takes place at 3 or 6 positions. The reactivity of 1 and 8 positions is probably prevented by the rigid structure of carbazole heterocycle.

In order to guarantee de oxidation of the carbazole core, and so, polymerization process, a minimum electronic density has to be ensured in the starting monomer. Consequently, strong electron-withdrawing substituents in the aromatic framework do not benefit the reaction.

**Figure 5:**
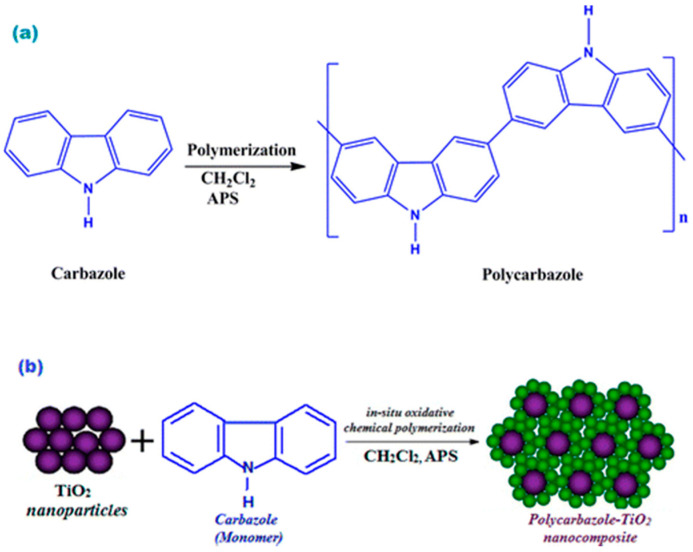
Schematic Diagram of the Formation Mechanism of (**a**) PCz and (**b**) PCz/TiO_2_ Nanocomposite. Reprinted with permission from Shakir et al. [100]. Copyright (2014) American Chemical Society.

Shakir and co-workers [100], reported one of the first attempt to obtain a new conductive nanocomposite of polycarbazole (PCz) with titanium dioxide (TiO_2_) nanoparticles. This composite was successfully synthesized by in-situ chemical polymerization in the presence of different amounts of nanosized TiO_2_ using ammonium persulfate (APS) as oxidizing agent, in 1:1 molar ratio (Cz:APS), in dichloromethane at room temperature for a period of 24 h (Figure 5). It is important to notice that, in a first step, the different solutions of TiO_2_ nanoparticles were added dropwise to the monomer solution under constant stirring, this step allows the carbazole absorption on the surface of the nanoparticles before its polymerization. Creamy coloured solutions were obtained which later transformed into greenish black sediments. The characterization results revealed that the polymerization of PCz had been achieved on the surface of the TiO_2_ nanoparticles indicating strong interaction between PCz and TiO_2_ nanoparticles. The same approach was also carried out by Baig et al. [101] for the synthesis of zirconium (IV) phosphate/polycarbazole nanocomposites.

It is important to notice that this nanocomposite presents antimicrobial properties. The antibacterial activity was in vitro evaluated against *Staphylococcus aureus*, *Staphylococcus epidermidis, Proteus mirabilis*, and *Escherichia coli.* Shakir et al. reported an improvement in the antimicrobial activity for the PCz/TiO_2_ Nanocomposite compared to TiO_2_. 

The first work on the chemical synthesis of unsubstituted polycarbazole and the formation of hollow microspheres based on this polymer was reported by Gupta and Prakash in 2010 [102]. Interfacial polymerization of carbazole was carried out using ammonium peroxodisulfate (1.2 M) as oxidizing agent in dichloromethane at room temperature. After 12 h of polymerization, dark green polycarbazole films were obtained with a yield of 50% ± 2%. During this interfacial polymerization, three-dimensional hollow spheres of polycarbazole of various diameters in the range of a few micrometers were obtained. The growth of these spheres was observed using scanning electron microscopy (SEM) and atomic force microscopy (AFM) techniques at different time intervals. These hollow microspheres are grown in the carbazole micelles formed in the interface. That is, monomer micelles are formed in the reaction solution at the interface due to the agitation (mechanical or thermal) [102,103]. The polymerization begins inside the micelles and they act as a template for the polycarbazole, being the size of the micelle dependant on the reaction conditions (temperature, concentration, stirring rate, etc.).

A similar procedure was followed by Sangwan et al. [99], in their work the effects of surfactants and their concentration on interfacial polymerization of carbazole was studied. Three surfactants of different nature such as nonionic Tween 20 (TW20), cationic hexadecyltrimethylammonium bromide (CTAB), and anionic sodium dodecyl sulphate (SDS) were used for different micelle formation. Ammonium persulfate (APS) was used as oxidizing agent, being the polymerization performed in dichloromethane (DCM) at 25 °C for 24 h. 

**Figure 6:**
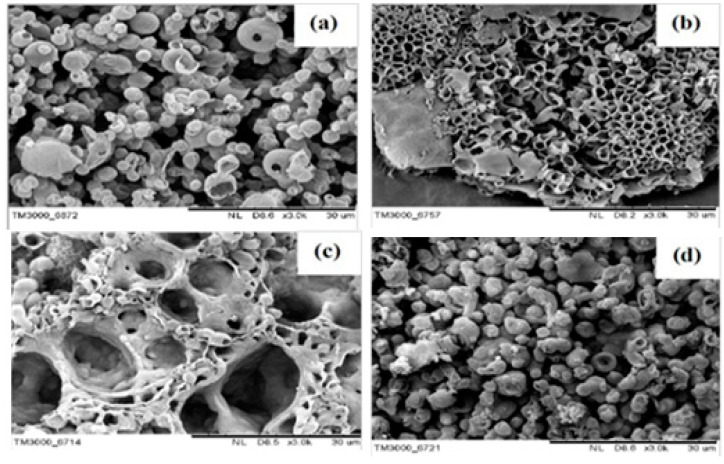
SEM images of the different PCz synthesized with different surfactant type: (**a**) PCz; (**b**) PCz/TW20; (**c**) PCz/CTAB; and (**d**) PCz/SDS at 24 h. Reprinted with permission from Sangwan et al. [99]. Copyright (2016) Wiley. The scale bar is 30 μm.

The reported SEM images revealed several PCz morphologies depending on the surfactant type and concentration. As it could be observed at Figure 6, macroporous honeycomb (Figure 6b), connected hollow spheres (Figure 6c), and smaller hollow spheres (Figure 6d) when using TW20, CTAB and SDS, respectively. On the other hand, for the system with no surfactant, the particle shapes are typically hollow sphere structures (Figure 5a). In addition, the electrical conductivity of the different PCz were measured being 1.72 ± 0.06 × 10^−4^, 2.62 ± 0.79 × 10^−3^, 2.16 ± 1.79 × 10^−5^ and 2.72 ± 0.32 × 10^−6^ S.cm^−1^, for PCz, PCz/TW20, PCz/CTAB and PCz/SDS, respectively. In this case, the maximum electrical conductivity was achieved for PCz/CTAB formulation. They observed that the electrical conductivity depends of the packaging capacity of the PCz particles. The particle size obtained was 3213 ± 944, 1182 ± 327, 2068 ± 455, and 2841 ± 835 nm for PCz, PCz/TW20, PCz/CTAB, and PCz/SDS, respectively. That is, a smaller particle size provides higher packing that provides higher surface area for electron transfer. PCz/CTAB presented the highest conductivity due its high packaging. However, in the case of neutral surfactant, even if the particle size was small, the surface seems to be not adequate to obtain good electrical conductivity compared to other formulations. Moreover, the materials were doped with HClO_4_ at different ratios. Overall, an important increase on the electrical conductivity was observed for all the materials. Moreover, it is important to notice the maximum value obtained for PCz/CTAB doped at 1:50 PCz:HClO4, in which the electrical conductivity reached 11.3 ± 0.36 S.cm^−1^. 

**Figure 7:**
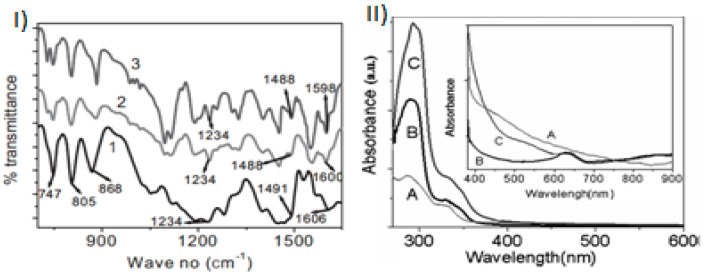
(**I**) FT-IR spectra obtained of (1) pure PCz (2) PCz–Au nanocomposite (by emulsion) and (3) PCz–Au nanocomposite (by interfacial polymerization). (**II**) UV–Visible spectra of (A) Pure PCz, (B) PCz–Au nanocomposite (by emulsion) and (C) PCz–Au nanocomposite (by interfacial polymerization). Inset: Zoom for small peak due to the gold particles (600–620 nm) and broad absorption band onward 750 nm. Reprinted with permission from Gupta et al. [104]. Copyright (2012) Wiley.

In 2011, Gupta et al. [104], reported the fabrication of PCz/gold nanoparticles nanocomposite by chemical synthesis using gold (III) chloride hydrate (HAuCl_4_) as an oxidizing agent by two different techniques: emulsion and interfacial polymerization. In the proposed mechanism, the synthesis of both PCz and gold nanoparticles undergoes in cooperation, Au^+3^ is reduced to Au^0^, whereas, the monomer is oxidized, that is, gold nanoparticles are formed simultaneously to the PCz polymerization. The product was obtained as a green powder with 85% and 75% yields for emulsion and interfacial polymerization, respectively, after 24 h of polymerization in dichloromethane. It is interesting to notice that UV–Vis spectra and Fourier-transform infrared (FTIR) spectra revealed the charge transfer between the polymer matrix and nanoparticles and interaction (Figure 7), indicating that this metal-polymer hybrid nanomaterial had improved technologically useful properties for molecular electronics system. 

In addition, others oxidizing agents, such as anhydrous ferric chloride (FeCl_3_), have been also used in the synthesis of PCz. Polycarbazole was synthesized with FeCl_3_ oxidation in chloroform at room temperature for 24 h. A green precipitate was collected, and washed in order to remove the Fe moieties, being the polymerization yield 80% ± 2% [105]. However, despite an intensive cleaning of the resulting product, iron moieties are still present in the polymer. The hypothetical interaction between iron and polymer could be based on two different assumptions. So, metallic moieties could be trapped in bulk polymer or cationic ions could be coordinated with the nitrogen of the carbazole. On the other hand, these moieties could affect positively to this polymer properties since this PCz presents higher affinity for proton that could be related to the presence of Fe ion. Due to this proton affinity, PCz with iron moieties could be used in sensors [105].

**Figure 8:**
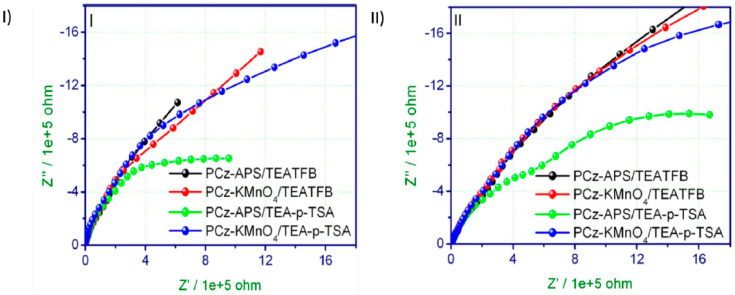
EIS responses, in form of Nyquist plots, for PCz–APS and PCz– KMnO_4_ in the presence of 0.1 m TEATFB and 0.1 m TEA-p-TSA in acetonitrile at different electrodes: (**I**) GC and (**II**) Pt. Reprinted with permission from Kumar et al. [106]. Copyright (2015) Wiley.

A comparative study in terms of the structural, thermal, morphological, and electrochemical properties of polycarbazole (PCz) synthesized by controlled interfacial polymerization using two different oxidizing agents, ammonium persulfate (APS) and potassium permanganate (KMnO_4_) has been reported by Kumar et al. [106]. The polymerization was carried out in the dark at room temperature in dichloromethane for 24 h, with good yields for PCz–APS and PCz–KMnO_4_, 82% and 75%, respectively. In this work, electrochemical impedance spectroscopic studies of both polymers were carried out in order to analyse their charge-transfer properties in the vicinity of modified PCz/glassy carbon (GC) and PCz/ Pt electrodes. Two supporting electrolytes were used in this study, namely 0.1 M tetraethylammoniumtetrafluoroborate (TEATFB) and 0.1 M tetraethylammonium-p-toluene sulfonic acid (TEA-p-TSA). Figure 8 shows the EIS response obtained for this study, in the form of a Nyquist plot. The comparison between the synthesized polymers indicates that PCz–APS presents better electron transfer kinetics compared to PCz–KMnO4 at either electrode (GC) or (Pt). 

### 2.2. Electropolymerization of Carbazole and Its Derivatives

**Figure 9:**
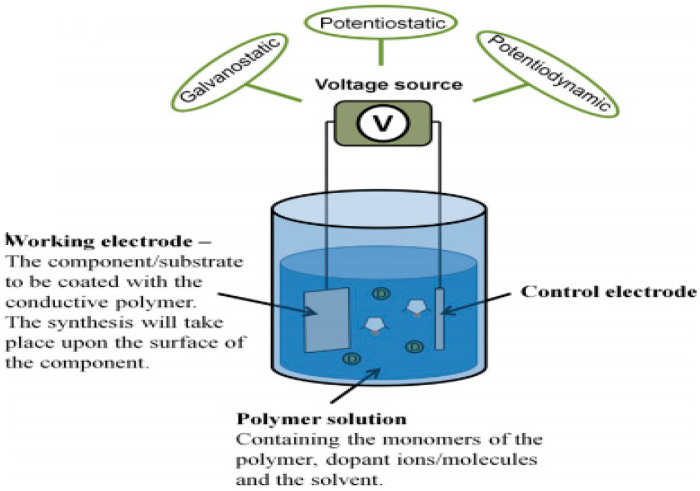
A schematic of the electrochemical synthesis set-up. Reprinted with permission from Balint et al. [114] Creative Commons CC-BY.

Conductive polymers could be directly synthesized in their doped conductive form from their monomer by an anodic or cathodic reaction. However, anodic polymerization is still the most widely used method. This method offers several advantages, it does not require the addition of catalyst in the electrolytic medium, so it could be considered a clean method and it does not require passage through a halogenated substrate (direct grafting of polymer on a substrate). Generally, this technique consists on the deposition of a polymer film by oxidation, that is, an anodic polymerization on the surface of an electrode of noble metal (gold, platinum) or other conductive materials such as glassy carbon or ITO (indium tin oxide) (Figure 9) [77,107,108].

On the other hand, cathodic polymerization is less implemented than the anodic oxidation method. It consists of two successive electrochemical reactions, followed by a chemical reaction that requires a catalyst such as nickel [109]. The material deposited on the electrode is obtained in the neutral state, therefore non-conductive, which could inhibit the reaction and requires to regenerate the active surface by doping the polymer [110,111,112].

Films obtained by electrochemical polymerization are films with better-defined and controlled properties and structure. The electrochemical polymerization has been widely used in recent years for the synthesis of insulating or semi-conductive polymers. This technique presents several advantages, such us homogeneity, relative ease of processing, and obtaining films with controllable and reproducible thickness and structure. It is also important to notice that these polymer thin films are usually difficult to prepare due to their low solubility in solvents [113].

Electrochemical oxidation of carbazole proceeds in a similar way to chemical oxidation but seems to be more selective. The first and most significant study on the electrochemical oxidation of carbazole was published by Ambrose and Nelsonin in 1968 [115].

Ates and Özyılmaz [49] conducted systematic study of corrosion performance of polycarbazole (PCz) and PCz derivatives. In their study, films of PCz, and two nanocomposites of nanoclay and zinc nanoparticles were developed. Films were chemically and electrochemically deposited on a stainless steel (SS304), and their anticorrosive properties were tested against 3.5% NaCl solution by EIS and potentiodynamic polarization curves. Carbazole was electropolymerized by chronoamperometric technique on an SS304 electrode for 3600 s in an oxalic acid/acetonitrile solution. In addition, the chemical polymerized carbazole was carried in acetonitrile using cerium ammonium nitrate (CAN) as initiator for 6–8 h at room temperature. This study showed that PCz, PCz / nanoclay and PCz / nanoZn films obtained using chemical method coated on SS304 electrodes displayed better corrosion protection performance compared to the films obtained by the electrochemical method. For chemically technique, PCz films, the highest protection efficiency (PE = 99.81%) has been obtained.

Srivastava et al. [51] reported PCz electropolymerization and deposition on ITO-coated glass. A study of polycarbazole films prepared on the different metal contacts, such as Aluminium, Copper, and Tungsten, was also carried out for the fabrication of Schottky diodes. PCz was synthesized by oxidative polymerization of carbazole in dichloromethane and as an oxidant tetrabutylammonium perchlorate (TBAP) in an electrochemical workstation. The polymerization of carbazole requires a low anodic potential (1.3 V) to be oxidized. The electrodeposition was prepared in a similar way. The metal contact was deposited in a previously fabricated PCz/Ito films by vacuum thermal evaporation deposition. Authors reported the fabricated diodes presented reasonably good performance rating parameters, showing the ITO/PCz/W device exceptionally good barrier height (0.95) and reverse saturation current density (J_0_) of 1.312 × 10^−13^ A/cm^2^.

To the best of our knowledge, only few samples of PCz and biopolymer composites have been reported until date. Kayan et al. [107] reported a study in which a polycarbazole/chitosan composite (PCz/Chi) films were successfully synthesized. The synthesis was carried out by using electrochemical polymerization by depositing on a Pt disk electrode by cyclic voltammetry after 5 cycles in the range of 0.0 V to +1.6 V in acetonitrile solution and 0.1 M lithium perchlorate as a supporting electrolyte. The composites were obtained by a similar procedure, adding solution of chitosan at different concentrations. Authors reported an increase the electrical conductivity of the films increase with the presence of chitosan. On the other hand, EIS measurements indicated that small amount of chitosan could enhance films conductivity by easing electron transfer.

### 2.3. Polymerization of N-Substitution Carbazoles

Poly(N-vinylcarbazole) (PVK) is one of the most interesting polymers based on N-substituted of carbazole due to its wide applications and its excellent thermal stability, doping behaviour, and UV durable property [77,107,108]. However, PVK present poor processability due to the π−π electron system along its backbone reducing its stability versus oxidation, which reduces the conductivity of the polymers [116,117]. In order to reduce these disadvantages, carbazole-based monomers have been modified. The substitution at N-position with a wide variety of functional groups could provide carbazole derivatives with improved properties such as solubility, better thermal stability, electrical, photoelectric, ion exchange and other physicochemical properties [118,119]. The improvement on their properties wides the applicability of these materials [120,121].

The polymerization N-vinylcarbazole (NVK) was reported for the first time by Reppe and co-workers in 1934 [122]. N-vinyl carbazole polymerization (NVK) is extensively investigated and many methods have been used, such as free radical [123], cationic polymerization [124], anionic polymerization [125], atom transfer radical polymerization (ATRP) [126], reversible additional fragmentation chain transfer (RAFT) polymerization [127], nitroxide-mediated polymerization (NMP) [128], charging transfer [129], electrocuting [129], solid state polymerization [130], and organometallic-mediated radical polymerization [131].

More recently, Marimuthu and Murugesan [132] reported an efficient and facile polymerization of N-vinyl carbazole (NVK). 1,4-bis (triethyl methyl ammonium) benzene dibromide (TEMABDB) was used as multi-site phase transfer catalyst (MPTC) and potassium peroxydisulphate (PDS) as water soluble initiator at 40 ± 2 °C in two phase system (cyclohexane/water) with ultrasound condition (45 kHz/550 W) and silent. The polymerization rate for this system was significantly increased when ultrasound was used.

**Figure 10:**
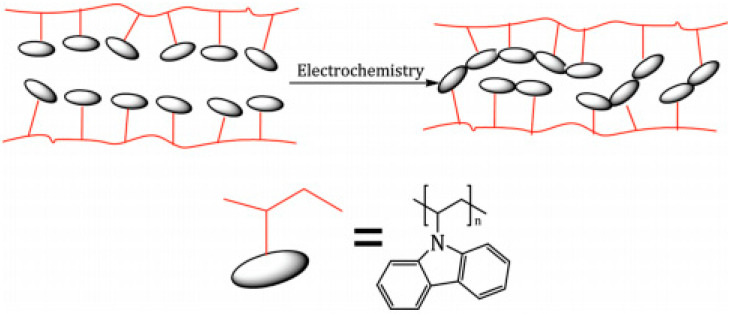
Schematic representation of the precursor polymer approach to CPN films. Reproduced with permission from Frau et al. [133]. Copyright (2010) American Chemical Society.

Frau et al. [133] reported the development of a conjugated polymer network (CPN) based on PVK to fabricate anticorrosion coatings. Electrochemical deposition on steel and ITO substrates by both potentiostatic and potentiodynamic methods were used. Anodic oxidation of the carbazole functional groups was used to prepare a cross-linked macromolecular structure (Figure 10). The electrodeposition of a PVK in dichlorometane was carried out on a potentiostat with a Pt wire as a counter electrode, nonaqueous Ag/AgCl electrode (0.1 M in acetonitrile) as a reference electrode, and, finally, steel or ITO films as working. A step of 1.2 V for 1000 s was induced for the potentiostatic deposition, whereas potentiodynamic deposition was carried out by cycling the potential between 0 and 1.4 V and a rate of 50 mV s^−1^ for 20 cycles. Morphological studies indicted a higher roughness on the substrates after a potentiostatic deposition compared to potentiodynamic deposition. In addition, the EIS results demonstrated that the PVK coating present good ion transport blocking properties, according to accelerated corrosion tests. Moreover, they showed efficient corrosion resistance on steel coupons used as a model metal substrate.

**Figure 11:**
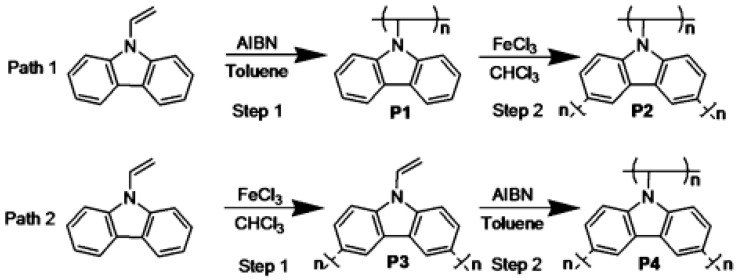
Scheme of the two synthetic routes described by Huang et al.: path 1 and path 2. Reproduced with permission from Huang et al. [134]. Copyright (2014) Royal Society of Chemistry.

Selection of the appropriate monomer and the right polymerisation techniques are crucial considerations for materials with excellent gas-uptake capacities. In this context, conjugated microporous polymers (CMP) have arisen as very promising type of microporous organic polymers (MOP). Huang et al. [134] reported two simple strategies (name in their work as path (1) and path (2)) for the preparation of CMPs based on N-vinyl carbazole derivatives. Four different derivatives (P1 to P4) were synthesised by combining both free radical polymerization and oxidative FeCl_3_ polymerization (Figure 11). The oxidative polymerization was performed at ambient temperature in chloroform using FeCl_3_ as oxidizing agent for 24 h. On the other hand, the free radical polymerization was carried out by using 2, 2′-azobisisobutyronitrile (AIBN) as initiator in toluene at 70 °C for 6 h. The effects of synthetic methods and sequences on the performance as CMP were evaluated. The BET surface area of the polymers was determined. In path 1, the BET obtained for P2 (878.46 m^2^ g^−1^) was significantly higher than that of P1 (68.65 m^2^ g^−1^). However, in path 2, the values obtained for both polymers were similar, being 621.18 m^2^ g^−1^ and 660.62 m^2^ g^−1^ for P3 and P4, respectively. The gas uptake results evaluated for the absorption of carbon dioxide (CO_2_), methane (CH_4_), and hydrogen suggested that P2 presents the best performance, that is, path 1 was the most appropriate method to obtain N-vinyl carbazole-based CMPs.

In recent years, graphene has attracted tremendous attention due to its properties and versatility, being used in ON and OFF for memory devices or conducting nanocomposites, among others [135]. Santos et al. [136] reported the preparation thin films of poly(N-vinylcarbazole)-GO (PVK-GO) nanocomposites via electrodeposition by cyclic voltammetry (CV) on bare ITO by repeatedly scanning the potential between 0 to 1500 mV at a scan rate of 10 mVs^−1^ for 50 cycles. The nanocoposite was crosslinked due to the electropolymerization process of the carbazole side groups of PVK. This improved the stability of the nanocomposites to several solvents such as 2-pyrrolidone and N-methylpyrrolidone, close to 30 days.

Similarly, Wang et al. [137] synthesized poly(9-vinylcarbazole)/silver nanocomposites by in situ formation of silver nanotubes and networks formed at the air–water interface via the reduction of Ag^+^ ions. The structures of the silver nanotubes were strongly dependent of the experimental conditions such as temperature.

**Figure 12:**
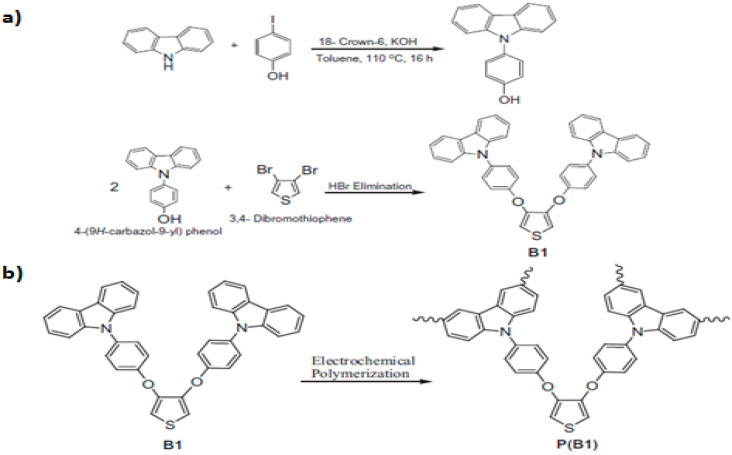
The performed polymer synthesis: (**a**) Synthesis of bis-4-(9H-carbazol-9-yl) phenyl-3,4-diyloxy thiophene (B1). (**b**) Electrochemical synthesis of P(B1). Reproduced with permission from [142]. Copyright (2015) Elsevier.

The stability of the redox states is the most suitable property for an electro-active polymer to be useful in building new electrochromic device [138,139,140,141]. Furthermore, the ability of a material to demonstrate a significant colour shift is important to electrochromic applications. Kocaeren [142] reported the synthesis of carbazole derivatives with electrochemical and electrochromic properties to be used electrochromic devices (ECDs). Firstly, bis-4-(9H-carbazol-9-yl) phenyl-3,4-diyloxy thiophene (B1) was synthesized from the reaction of 4-(9H-carbazol-9-yl) phenol and 3,4-dibromo thiophene in the presence of potassium carbonate (K_2_CO_3_) in tetrahydrofuran (THF). After that, the polymer of B1 was deposited onto an ITO-glass surface by oxidative electrochemical polymerization (Figure 12). The electropolymerization was performed on a potentiostat in acetonitrile, using Pt wire as counter electrode, Ag wire as reference electrode and ITO as working electrode, scanned from +0.3 to +1.4 V. The presence of the polymer was evidence due to the increase of a peak in a cyclic voltammetry at 0.95 V. It is important to notice that the polymer film presents a blue colour between 1.0 and 1.4 V due to its oxidation, whereas its colour turns into a light yellow between 0.5 and 0.9 V owning to its reduction. The maximum absorbance wavelengths were 320 and 670 nm. This carbazole derivative, which presents high stability, could be used in electrochromic devices (ECDs) according to redox stability measurements.

**Figure 13:**
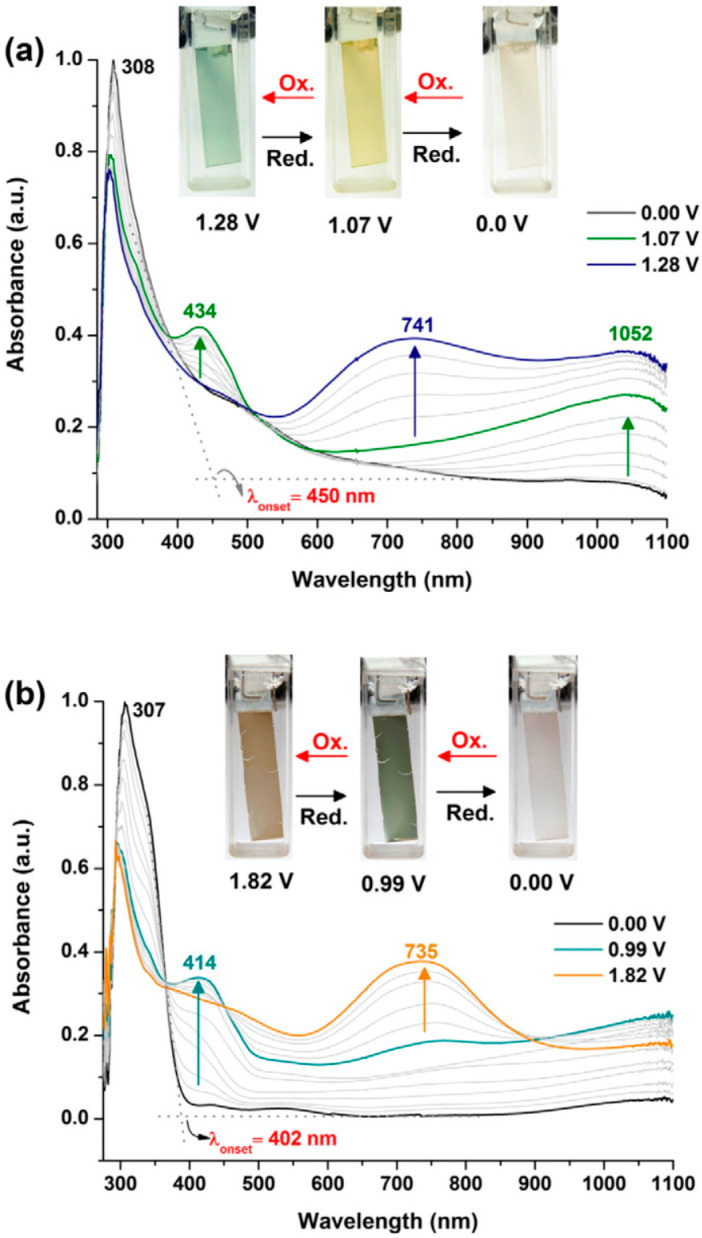
Electrochromic study of the (**a**) P(PhCz-2Cz) and (**b**) P(TPA-2Cz). Reproduced with permission from [143]. Copyright (2015) Elsevier.

Another example of electrochromic carbazole derivative was synthetized by Hsiao and co-worker [143]. In their study, two previously synthesized carbazole-based monomers were successfully electrodeposited and polymerized onto the ITO electrode by electropolymerization. 4,4′-di(carbazol-9-yl)-4″-methoxytriphenylamine (TPA-2Cz) and 3,6-di(carbazol-9-yl)-N-(4-methoxyphenyl)carbazole (PhCz-2Cz). The electropolymerization onto an ITO (working electrode) of the monomers was carried out with a polymer and 0.1 M Bu_4_NClO_4_ solution in dichloromethane and ITO as working electrode by several cycles between 0 and 1.4 V at a scan rate of 50 mV s^−1^. P(PhCz-2Cz) (Figure 13a) present a blue-green colour in its maximum oxidation state around 1.28 V. This film changes to yellow (1.07 V) and finally, colourless at 0.0 V. Similarly, P(TPA-2Cz) presents a brown colour at its fully oxidized state and dark green colour at semi-oxidized states.

The same strategy was used in other work of Hsiao and Lin [141] in which poly(amide-carbazole) and poly(imide-carbazole) were used for the development of two series of diamide-cored carbazole dendrons (6CzR-DA) and diimide-cored carbazole dendrons (6CzR-DI). These monomers series were synthesized from condensation reactions of 3,6-di(carbazol-9-yl)-N-(4-aminophenyl)carbazole (NH_2_-3Cz) with aromatic dicarboxylic acids and tetracarboxylic dianhydrides, respectively. Similar to their previous work, these polymer films showed good electrochemical and electrochromic properties.

**Figure 14:**
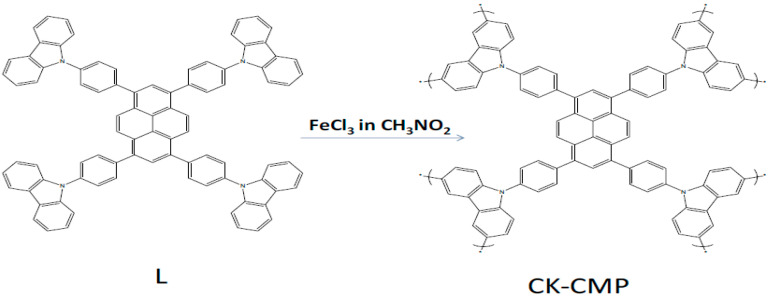
The synthesis of CK-CMP proposed by Yang et al. in their work. Reproduced with permission from [144]. Copyright (2019) Elsevier. L: 1,3,6,8-tetrakis(4-(9H-carbazol-9-yl) phenyl)pyrene, CK-CMP: conjugated microporous polycarbazole derivative.

Yang and co-workers [144] performed a study on induced oxidative polymerization of 1,3,6,8-tetrakis(4-(9H-carbazol-9-yl) phenyl)pyrene (L) with FeCl_3_ as oxidant in anhydrous chloroform for 24 h at 60 °C. This process resulted in the formation of the bulk polymer (Figure 14), being a highly luminescent conjugated microporous polycarbazole derivative (CK-CMP).

**Figure 15:**
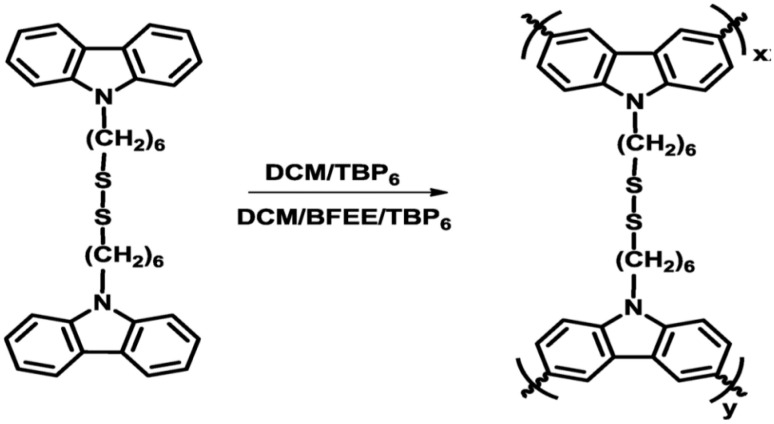
Electrochemical polymerization of 1,2-bis[6-(9-carbozol-9-yl)hexyl]disulfane proposed by Soganci et al. Reproduced with permission from [48]. Copyright (2018) Elsevier.

Soganci et al. [48] reported a novel method for electropolymerization a disulfide-linked N-alkyl substituted carbazole derivative. 1,2-bis[6-(9-carbozol-9-yl)hexyl]disulfane (CS) monomer was synthetized in this work (Figure 15) and then, electropolymerized using cyclic voltammetry and ITO as working electrode. The electropolymerization process of CS monomer was performed comparatively in the BFEE (Boron trifluoride diethyl etherate) containing solution and BFEE-free electrolytic solutions. This material presents interesting electrochromic properties that could be potentially used in smart window applications, due to the high optical contrast value and stability obtained in BFEE compared to other N-alkyl substituted carbazole appeared in literature.

Duran et al. [145] successfully deposited a poly(N-methyl carbazole) (PNMeCz) coating on stainless steel type 304. The film was deposited by electropolymerization of N-methyl carbazole (NMeCz) monomer in acetonitrile solution containing tetrabutylammonium perchlorate using cyclic voltammetry and stainless steel as working electrode. The film was electrodeposited applying a potential between +0.5 and +1.7 V with a rate of 50 mV/s during 15 cycles. The resistance to the corrosion was evaluated, demonstrating these films presented good anticorrosion properties.

**Figure 16:**
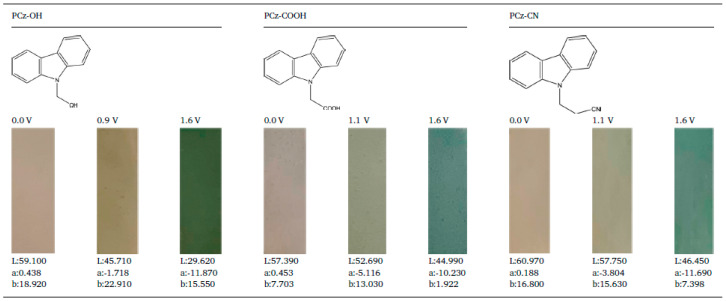
The colour variations induced by redox process on PCz−OH, PCz−COOH and PCz-CN. Reproduced with permission from [146]. Copyright (2020) Elsevier.

Elkhidr and co-workers [146], synthesized and studied different electrochemical and optical properties for three carbazole derivatives with different substitution at N-positions, methanol (carbazol-9-yl-methanol), carboxylic acid (carbazol-9-yl- carboxylic acid) and cyanoethyl (carbazol-9-yl-cyanoethyl). Polymeric films of these derivatives were obtained (PCz−OH, PCz−COOH, and PCz-CN, respectively) by the electropolymerization on ITO substrate by repetitive cyclic voltammetry. The electropolymerization was carried out on a potentiostat-galvanostat system, with tree electrodes; the working electrode was ITO, whereas platinum and silver wires were used as counter and pseodoreference electrodes, respectively. A potentiodynamic electropolymerization was performed between +0.0 to +1.6 V with a scan rate of 100 mV/s. The monomer solutions containing NaClO_4_-LiClO_4_ electrolyte dissolved in acetonitrile. Polycarbazole presents high solubility problems, being almost insoluble in most of inorganic solvent and soluble in only few organic solvents. However, the polycarbazole derivatives reported in this work showed a good solubility in common organic solvents such as dimethyl sulfoxide (DMSO), N-methyl-2-pyrrolidone (NMP), tetrahydrofuran (THF), and dimethylacetamide (DMAC). The colour variations induced by the redox switching of the carbazole derivatives during the electrochemical process are summarized in Figure 16, where L is the luminance or brightness, a is hue and b is the saturation using the International Commission on Illumination (CIE) system.

**Figure 17:**
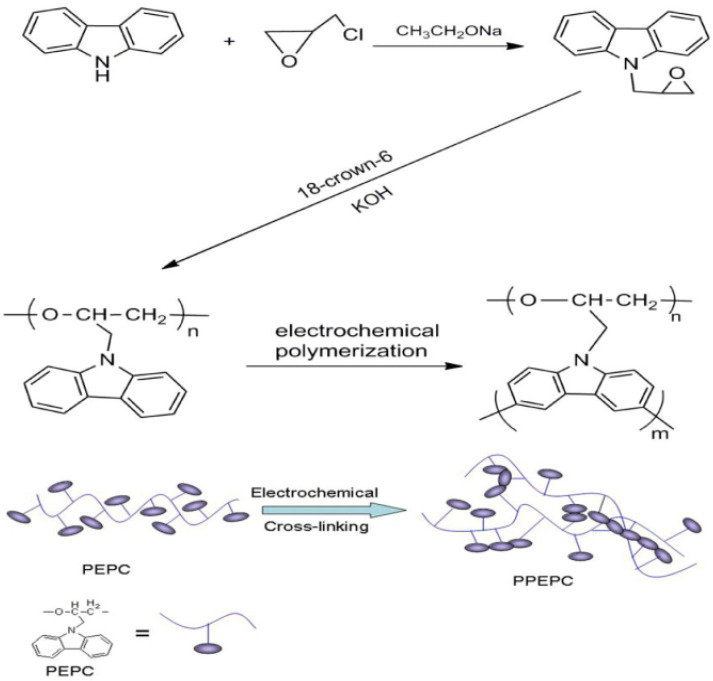
Synthesis route and scheme for PEPC and PPEPC. Reproduced from [147]. (CC by 4.0).

Qin et al. [147] reported the successful synthesis of conjugated network based on poly(ethylenoxide) grafted carbazole. In their study, first poly(N-epoxypropyl carbazole) (PEPC) was obtained by anionic ring-open polymerization of N-epoxypropyl carbazole (EPC) using potassium hydroxide and 18-crown-6 in toluene at 90 °C for 12 h. After obtaining PEPC, a conjugated network was fabricated by electrodeposition of poly[poly(N-epoxypropyl carbazole)] (PPEPC). The electropolymerization was carried put in a potentiostat using Pt wire as counter electrode and stainless steel and Pt sheets as working electrodes on which the polymer was deposited. A scheme of the complete synthetic process carried out in this work is depictured in Figure 17. The synthesized PPEPC showed favourable thermal stability and strong mechanical properties, and can be easily bent or cut into different forms.

## 3. Application

The carbazole based molecules have emerged as important core fragments with highly interesting biological activity. Its derivatives present several advantageous properties such as antimicrobial, antitumor, antioxidant, anti-inflammatory, and pancreatic lipase inhibition properties [43]. In addition to biological activities, these molecules also present opto-electronic, electrochemical, and electrical properties when polymerized, being their main applications such as photovoltaic devices, electroluminescent displays, batteries, bio(sensors), etc.

PVK presents adequate levels of photoconductivity to be used is electrophotography, being used by IBM in its Copier I series in 1970. In addition, poly(N-vinylcarbazole) has been widely used as a host material in OLEDs. PVK presents a high energy blue-emissive singlet excited state and could acts as electron donor and hole transporting material. However, since it is not a conjugated polymer its transport is carried out by radical cation hopping among the discrete carbazole units [77]. Since them, many research have been focus on the development of 3,6 disubstitued carbazoles, taking advantage of the high reactivity of 3,6 positions [148]. In this context, linear and hyperbranched 3,6-carbazole derivatives present excellent redox activity, photorefractive and nonlinear optical properties [106,149]. These properties make them good candidates as materials for organic light-emitting diodes (OLEDs). However, as has been previously mentioned, they present several limitations such as their reduced conjugation of electrons and low molecular weight. On the other hand, 2,7-carbazole-based polymers are usually more linear, this improves their organization and the extension of the conjugation length, construing to a lower band gap [150]. These properties are especially interesting for the development of organic field effect transistors (OFETs), bulk-heterojunction (BHJ) solar cells, thermoelectric [84,149].

It is important to notice that synthetic pathway could condition the potential applications. That is, chemical synthesis is more interesting for those applications in which bulk materials are needed, or a control over the morphology is required. In addition, the electropolymerization is more suitable to planar applications in which a coating or a thin film is required [102,151].

In this section, some of the last applications of above-mentioned potential fields of N-carbazole containing polymers are described.

### 3.1. Light Emitting Diode Application

**Figure 18:**
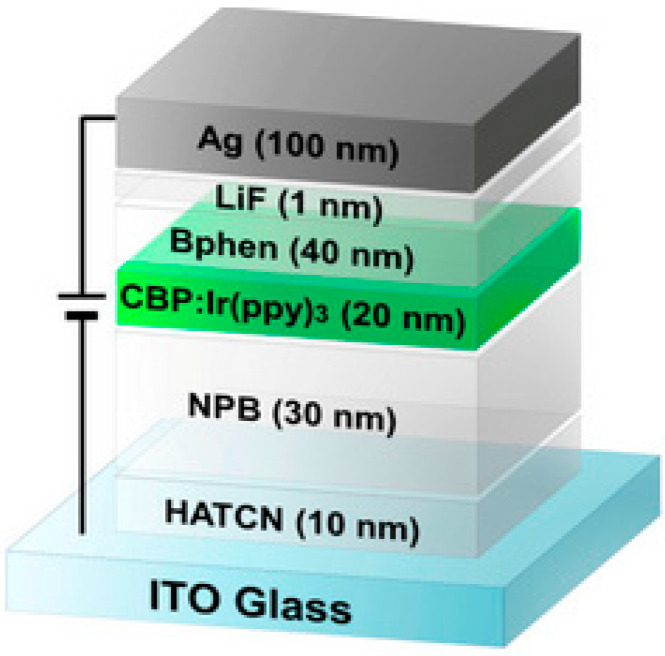
Example of a cross section of a polymer LED. Modified from [152] MDPI 2014 (CC by 3.0).

Organic light emitting diodes (OLEDs) made from polymers have attracted increasing interest since the group led by Richard Friend in 1992 [37] reported the emission of light from a semiconducting polymer sandwiched between two contacts and connected to a battery. In the Figure 18, an example of an OLED system could be observed [152]. OLEDs are arising as one of the most used technologies in full-colour displays and as an environmentally friendly lighting source. The present excellent colour quality, and they are cost-effective and mercury-free [153].

One to the first examples of PVK based OLED was reported by Hebner et al. [154]. They fabricated OLEDs with low turn-on voltages by ink-jet printing using PVK luminescence films with dyes coumarin 6. Low-molar-mass carbazole-based derivatives for organic light emitting diodes have been reviewed by Krucaite and Grigalevicius [155] in a recent study. They concluded that carbazole based materials could be used as hole transporting materials, emitting materials, thermally activated delayed fluorescence emitters and host materials for phosphorescent dopants and organic light emitting diodes.

A polymer used as light-emission material must meet two basic characteristics, electrical conductivity (semiconductive polymer) and high photoluminescence (PL) efficiency [156]. Among these materials, carbazol-based materials benefit from the wide bandgap of carbazole as well as its remarkable thermal, photochemical, and chemical stability. Especially, the relatively high triplet energy level of carbazole makes it an appealing candidate to design hosts for wide bandgap triplet emitters such as blue dopants [157]. In addition, the substituted carbazoles could cover the range of visible lights from blue to green colour due to the modification of the carbazole backbone with different moieties. For example, the introduction of 3(9)-aryl carbazoles and 3,6-diaryl substituted derivatives were very effective as the host materials for blue (EQE < 24%), green (EQE < 20%) and red (EQE < 19%) phosphorescent organic light emitting diodes [155].

Syutkin et al. [158] reported the synthesis of chalcones with carbazole substituents, being some of them capable to react with guanidine sulphate to produce 2-amino-4,6-diarylpyrimidines. This compound could form a stable coloured conjugated polymer films on the surface of a working electrode under conditions of cyclic voltammetry, being promising materials for the design of light emitting diode.

**Figure 19:**
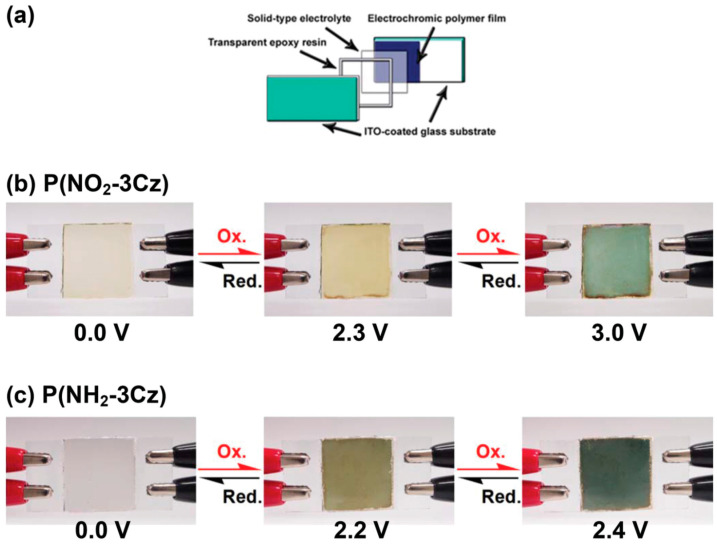
(**a**) Schematic illustration of the structure of the electrochromic devices. Sandwich-type devices, using (**b**) P(NO_2_-3Cz) and (**c**) P(NH_2_-3Cz). Reproduced with permission from [141]. Copyright (2016) Royal Society of Chemistry.

Hsiao et al. [141] reported the construction of electrochromic devices based on carbazole derivatives (poly(3,6-di(carbazol-9-yl)-N-(4-nitrophenyl)-carbazole) (P(NO_2_-3Cz) and poly(3,6-di(carbazol-9-yl)-N-(4-aminophenyl)carbazole) (P(NH_2_-3Cz)) electrodeposited on ITO substrates. In their study, single-layer electrochromic cells were fabricated, sandwich-type device (Figure 19a), to evaluate the potential application of this films in electrochromic devices. The sandwich type device was fabricated with a gel electrolyte spread on polymer-coated ITO substrate on side of the electrode, being closed by electrodes. In addition, the possible leakages were prevented by applying epoxy resin seal. The P(NO_2_-3Cz)-based device presents a slightly yellowish colour in its neutral form (0.0 V), and the intensity of the colour increased until darker yellow when the potential vas increased until 2.3 V (Figure 19b). However, the films turned in to a blue colour when the potential reached +3.0 V. The reversibility of this behaviour was successfully demonstrated, achieving the initial light-yellow colour when the potential was removed. On the other hand, the colouring and the potential peaks of the P(NH_2_-3Cz) film were slightly different depending on the substitution (Figure 19c).

### 3.2. Electrochemical Capacitors

Supercapacitors are considered as an energy storage system, electrochemical energy in this case, with an important future as an alternative to other classical methods. Considering their power and energy density, these can be located between batteries and traditional dielectric capacitors [159].

According to energy storage mechanism and electrode materials, the main electrochemical capacitors (EC) could be categorized in three families: carbon-based ultracapacitors, metaloxides-based supercapacitors, and conductive polymers based supercapacitors [160]. Polymer-based capacitators could be considered as crucial materials for the development of many high-power electrical systems [161]. Conductive polymers present good electrical conductivity, large specific power, simple synthesis and low cost. This properties have increase the interest on these materials as a promising materials for capacitors compared to expensive and toxic metal oxides, and carbon-based electrode materials [162,163,164]. Among these conductive polymers, carbazol-based polymers are often complementary parts of the active electrode material in capacitors. That is, when an electric field is applied ions are transfer in and out of the polymer backbone from the electrolyte over the course of the redox process [165]. This process occurs due to excellent attributes of hole transport, relatively high specific capacity, excellent atmospheric stability, in addition to their physical and electronic properties, such as surface morphology, thickness, electrical conductivity, internal resistance, and durability, which directly affect the performance of super capacitors [166]. However, the redox reaction could reduce the stability of the polymer inducing its degradation [167].

Ates and Uludag [168] reported the synthesis and study of the properties of a capacitator based on poly(9H-Carbazole-9-Carbothioic Dithioperoxyanhydride) (P(2CS2Cz)). P(2CS2Cz) was electrodeposited on a glassy carbon electrode (GCE) by cyclicvoltamentry. After the deposition, the capacitance of the films at different concentrations were evaluated, being the low frequency capacitance 0.52 mF cm^−2^ ([2CS2Cz] = 0.25 mM) and double layer capacitance 571 µF ([2CS2Cz] = 1 mM).

Wang et al. [169], have synthesized three porous polycarbazole networks from poly(4,6-tri(9H-carbazol-9-yl)-1,3,5-triazine) (PTCT), poly(4,4′,4″-tri-9-carbazolyltriphenylamine)(PTCA), and poly(4,4′-bis(9H-carbazol-9-yl)biphenyl) (PBCP) by a chemical oxidative polymerization. The capacitive properties and their capacity for CO_2_ storage were also studied. These polymers present a charge/discharge rate of 8 s that could be considered as a fast, and a electrochemical capacity of 558 F g^−1^. In addition, the porous network synthesized in this study presents a nanometric pore size, around 1nm, and large surface area, 1280 m^2^ g^−1^. These characteristics, added to their nitrogen-rich structure, enables an efficient CO_2_ capture, being of 20.4 wt% at 1 bar and 0 °C. The use of carbazole based materials as conjugated microporous polymer was also reported by Huang et al. [134].

Recently, Duran et al. [170] studied the supercapacitive performance poly(carbazole) films electrodeposited on stainless steel (SS) by using 0.1 M supporting electrolyte of lithium perchlorate (LP), sodium perchlorate(SP) and tetrabutylammonium perchlorate (TBAP) in acetonitrile to form PCz(LP), PCz(SP) and PCz(TBAP), respectively. The specific capacitance values obtained for the SS/PCz(LP), SS/PCz(SP) and SS/PCz(TBAP) systems were 133, 64, and 9 F g^−1^, respectively. Authors claimed that PCz(LP) could be considered as adequate material for supercapacitor applications.

### 3.3. Biosensor Applications

**Figure 20:**
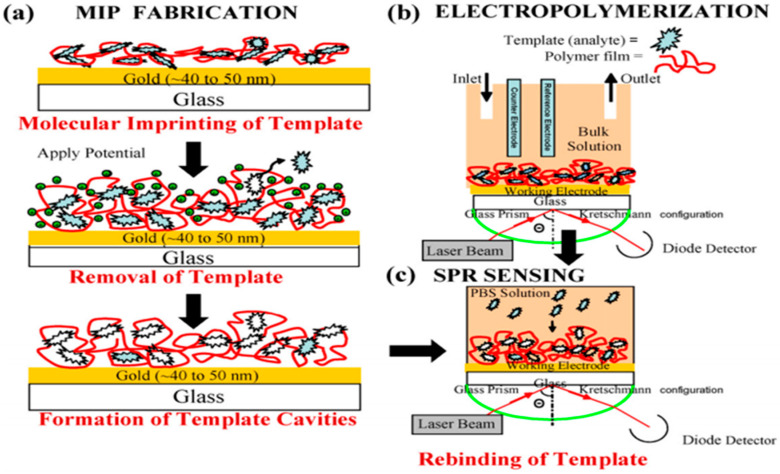
(**a**) Sensor film fabrication by molecular imprinting and template removal by constant potential wash at 0.4 V (versus Ag/AgCl). (**b**) ESPR in situ set-up for electropolymerization and and (**c**) SPR sensing of the imprinted guest molecule using. Reproduced with permission from [53]. Copyright (2011) Elsevier.

Conducting polymers have also successfully employed in biosensor applications [171]. Pernites et al. [53] developed a novel chemosensitive ultrathin films based on carbazole derivatives. The sensoring devices were based on electropolymerized molecular imprinted polymers (E-MIP) capable to detect three drugs, naproxen, paracetamol, and theophylline. The molecular imprinting enables a tailor-made specificity. In this case, the sensor is capable to recognize the selected drugs with a high selectivity, being in situ evaluated by surface plasmon resonance (SPR). Among, the different monomers used in this study author reported that bifunctional monomers containing –COOH and –OH functional groups were most effective for the imprinting process. Moreover, authors claimed that this method could be a promising approach for sensors fabrication. Figure 20 summarizes the E-MIP based sensor fabrication reported in this study.

**Figure 21:**
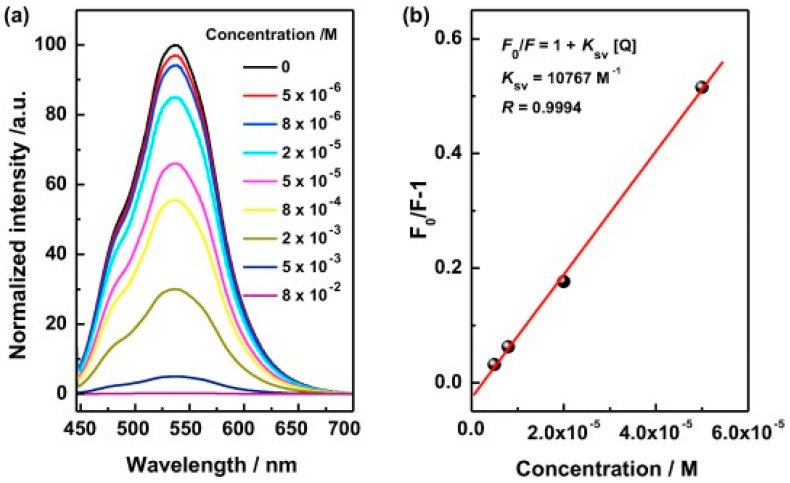
(**a**) Study of fluorescence emission quenching of PAC/ethanol dispersion at different nitrobenzene/ethanol solutions; (**b**) Stern-Volmer plot between F/F_0_ and nitrobenzene concentration. Reproduced with permission from [172]. Copyright (2019) Elsevier.

Novel fluorescent and conductive hollow microspheres based on aniline and carbazole derivatives were successfully synthesized by Chenga and co-workers [172]. The poly(aniline-co-3-amino-9-ethyl-carbazole) (PAC) obtained by oxidative copolymerization with APS was used as effective fluorophore. The sensoring capacity of the materials were evaluated for two types of analyte, namely nitro-based explosives and Cu^2+^ cations, with the reported detection limits of the material being 5 μM and 5 nM, respectively. Tested analytes decreased the fluorescence with the increase of their concentration, as can be observed in Figure 21 where the results obtained for nitrobenzene detection are shown.

**Figure 22:**
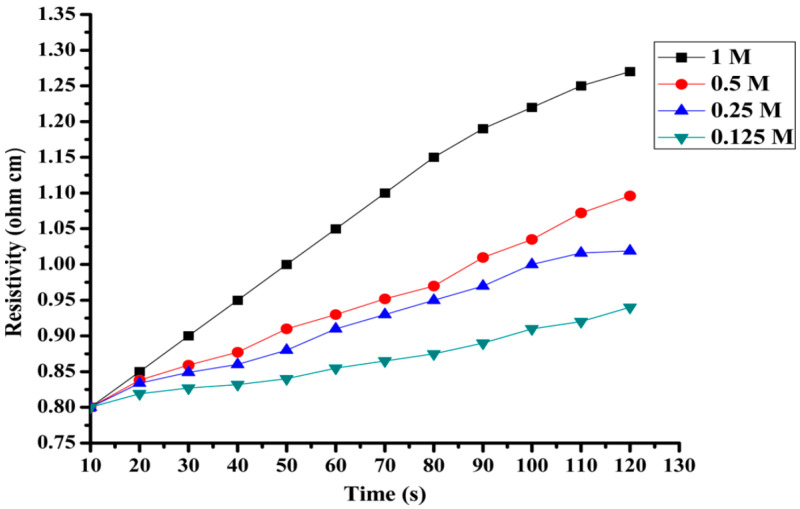
Effect on the resistivity of PCz/TiO_2_ nanocomposite on exposure to different concentrations of ammonia with respect to time. Reprinted with permission from Shakir et al. [100]. Copyright (2014) American Chemical Society.

Shakir et al. reported the development of PCz/TiO_2_ nanocomposite with good antibacterial activity, which presents also good capability for sensing ammonia. The evaluation of its capability for ammonia sensing was carried out by measuring resistivity changes on exposure to ammonia vapours. The nanocomposite showed a relatively fast response toward aqueous ammonia in the range of 0.25–1 M at room temperature (Figure 22).

### 3.4. Photovoltaic Devices Applications

Solar cells or photovoltaic devices capable to convert the solar energy into electric energy are under continuous development pushes by the need of greener electric sources. In this context, Li et al. [54], in addition to other authors [173,174,175,176,177], described in their review that carbazole-based polymers and copolymers could be considered among the most promising materials for highly efficient organic solar cells.

Fujita and Michinobu [178] synthesized a carbazole-based conjugated polymer as donor–acceptor type alternating copolymers. Different type of poly(1,8-diethynylcarbazole) were synthesized and their fluorescence and electrochemical properties were evaluated being adequate to be used as a p-type semiconductor in solar cells. Synthesized poly(1,8-carbazole)s were used in the preparation of bulk-heterojunction photovoltaic cells, photoconversion efficiency (PCE) of the cells was between 0.05% and 0.24%. Similarly, Qin and co-workers [179] synthesized a series of conjugated polymers with carbazole as the donor unit or benzothiazole as the acceptor unit to be used in solar cells presenting a PCE between 5.8% and 0.43%.

### 3.5. Memory Device Applications

In addition to the other possible applications, conducting polymers could be also used in the development of advanced memory devices due to their donor-acceptor (D-A) properties [136,180]. These D-A polymers present an electrically bistable behavior, this characteristic being highly desirable for memory devices. In addition, they present other characteristics that make them highly interesting, tailor-made structures, namely low-cost, processability (mainly in solution), and three-dimensional stacking capability [181]. In donor−acceptor (D-A) conjugated polymer systems, the charge transfer between donor and acceptor moieties could be controlled by the strength and ordering of donor and acceptor groups. These parameters play a critical role in enhancing electrically bistable switching behavior [182,183]. The most efficient way to enhance the intrinsic local packing of materials is to increase the planarity of the polymer main chain, which in turn can generate tight p–p stacking, because the fused ring structures are much flat than the conjugated ‘‘single’’ bond [184]. In general, the incorporation of different electron acceptors into conjugated polymeric donors significantly affects the memory properties [185]. The p-type conductive polymers could be employed as the donor materials and then they are mixed with a kind of commercial acceptor material [176]. Among them, the poly(N-alkyl-2,7-carbazole)s based D-A copolymers presents wide potential owning to their excellent hole-transporting property and good stability of the carbazole units [186].

**Figure 23:**
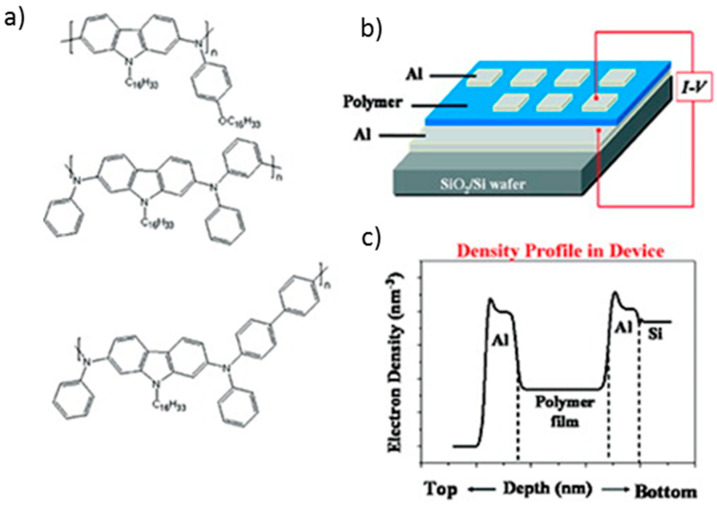
(**a**) Structure of poly(2,7-carbazole) derivate polymers obtained by Hahm et al.; (**b**) Scheme diagram of the memory devices fabricated and (**c**) obtained electron density profiles. Reproduced with permission from [187]. Copyright (2011) American Chemical Society.

Hahm et al. [187] synthesized several poly(2,7-carbazole) derivatives (Figure 23) and successfully studied their electrical memory properties. In order to evaluate their memory characteristics, a memory device was fabricated (Figure 23). First, aluminium was deposited on glass substrates by electron beam sputtering, then the different polymer substrates were deposited and, finally, additional aluminium electrodes were deposited on the top thermal evaporation. All tested polymers/aluminum sandwich type devices presented similar dynamic random-access memory (DRAM). They present ON/OFF current ratios between 10^5^ and 10^9^. Considering the obtained results these materials could be suitable for the production of low-cost programmable DRAM devices with a high-performance and capable to operate under very low power consumption.

Similar system was reported by Zhang and co-workers [188]. In their study, they described a write-once, read-many-times (WORM) memory effect in sandwich type devices fabricated by using different carbazole derivatives, aluminum, and ITO. These poly(carbazole)-based polymers present a characteristic donor–trap–acceptor (D-T-A) structure and an electrical bistability due to the field-induced charge-transfer interactions. The results indicated that the devices could present a non-volatile non-erasable memory behaviour.

**Figure 24:**
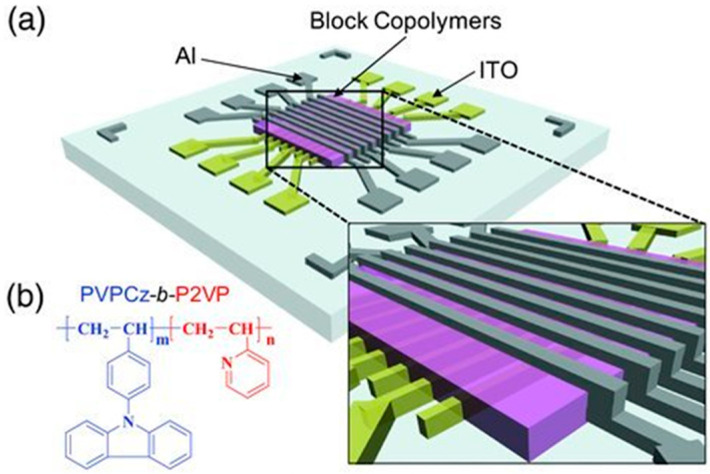
(**a**) A Scheme of the fabricated organic memory device (Al/polymer/ITO); (**b**) Structure of the block copolymer of PVPCz-*b*-P2VP. Reproduced with permission from [189]. Copyright (2011) Wiley.

Structural and electrical characterization of a block copolymers based unipolar non-volatile memory device were fabricated by Kang et al. [189]. In this study, a crucial factor was to adjust the block ratio of poly(9-(4-vinylphenyl)carbazole)-*b*-poly(2-vinylpyridne) (PVPCz-*b*-P2VP) copolymer in order to present a lamellar structure that could induce a unipolar switching behavior. This switching property was highly interesting for the fabrication Aluminum/PVPCz-*b*-P2VP/indium tin oxide (ITO) based memory devices (Figure 24). The memory behavior studies of these devices revels a good endurance cycling, around to 190 cycles, and a high ON/OFF ratio, higher than 10^4^.

## 4. Conclusions and Future Perspective

Polycarbazole and its derivatives have been extensively used for long time. This review describes the synthesis and applications of carbazole-based materials on the last 10 years. However, as can be observed by the increase in the number of publications in the field of conductive polymers, there is an increasing demand for conductive polymers in several applications, such as light emitting diodes (OLEDs), capacitators, or memory devices, among others. This demand has renewed the interest on carbazole-based materials.

Considering the synthesis of carbazole-based polymers, the electrochemical polymerization has revealed the most employed methodology since many applications requires of a thin film of conductive polymer and this technique is highly suitable for film development. On the other hand, several research works have focused their attention to the development of new carbazole-based monomers with complex structures in order to improve or tailor the properties of these materials, and thereby increasing their potential applications. The variation in the structure of the carbazole derivatives could improve their processability and solubility, but also increase the conjugation length of the electrons through their structure, or vary their opto-electrical properties. In this context, it is important to highlight the emergence of flexible and wearable electronic devices as a part of the internet of the things (IoT). In fact, the carbazole-based materials could have a promising future. The need for conductive polymers capable to respond adequately to highly demanding requirements (mechanical or conductive, among others) could encourage the development of new carbazole-based materials.

Induced by the emergence of the internet of the things, flexible and wearable electronic devices have attracted great interest in the last years. These devices have gradually emerged in daily life due to their lightweight, the ability to attach onto clothes, or easy skin attachment potential, and their ability to withstand mechanical deformation. Among their applications, one could highlight portable displays, human activity monitoring sensors, and self-powered devices. The high physicochemical requirements, such as high electrical conductivity, good tensile strength, high flexibility, and light weight, fast oxidation/reduction reaction kinetics have distinguished CPs as excellent candidates. Carbazole based polymers are promising for constructing flexible energy harvesting and storage devices.

## Figures and Tables

**Table 1:** Summary of the reviews on poly(carbazole)derivatives extracted from Scopus.

Authors	Title	Year	Journal	Cited	Ref.
Houben J.L. et al.	Optically active vinyl polymers containing fluorescent groups: 5. Fluorescence properties of poly(9-vinyl carbazole) and optically active polymers containing carbazole units	1978	Polymer	35	[75]
Murphy S.M. et al.	Polymer membranes in clinical sensor applications. II. The design and fabrication of permselective hydrogels for electrochemical devices	1992	Biomaterials	27	[76]
J.V. Grazulevicius et al.	Carbazole-containing polymers: synthesis, properties and applications	2003	Progress in Polymer Science	660	[77]
Morin J.-F. et al.	Polycarbazoles: 25 years of progress	2005	Macromolecular Rapid Communications	552	[74]
Ding J. et al.	Highly efficient green-emitting phosphorescent iridium dendrimers based on carbazole dendrons	2006	Advanced Functional Materials	290	[78]
Faridbod F. et al.	Developments in the field of conducting and non-conducting polymer based potentiometric membrane sensors for ions over the past decade	2008	Sensors	120	[79]
Boudreault P.-L.T. et al.	Poly(2,7-carbazole)s and related polymers	2008	Advances in Polymer Science	61	[71]
Zou Y., Gendron D. et al.	A high-mobility low-bandgap poly(2,7-carbazole) derivative for photovoltaic applications	2009	Macromolecules	220	[80]
Ates M. et al.	Conducting polymer coated carbon surfaces and biosensor applications	2009	Progress in Organic Coatings	106	[81]
Beaupré S. et al.	Solar-energy production and energy-efficient lighting: Photovoltaic devices and white-light-emitting diodes using poly(2,7-fluorene), poly(2,7-carbazole), and poly(2,7-dibenzosilole) derivatives	2010	Advanced Materials	187	[82]
Boudreault P.-L.T. et al.	Polycarbazoles for plastic electronics	2010	Polymer Chemistry	149	[70]
Dubey N., Leclerc M.	Conducting polymers: Efficient thermoelectric materials	2011	Journal of Polymer Science, Part B: Polymer Physics	252	[83]
Gendron D., Leclerc M.	New conjugated polymers for plastic solar cells	2011	Energy and Environmental Science	240	[84]
Grigoras A.G.	A review on medical applications of poly(N-vinylcarbazole) and its derivatives	2016	International Journal of Polymeric Materials and Polymeric Biomaterials	2	[85]
Tan S.E., Sarjadi M.S.	The recent development of carbazole-, benzothiadiazole-, and isoindigo-based copolymers for solar cells application: A review	2017	Polymer Science—Series B	7	[86]
Liguori R. et al.	Stereoregular polymers with pendant carbazolyl groups: Synthesis, properties and optoelectronic applications	2018	Synthetic Metals	2	[87]
Ghorbani Zamani F. et al.	Current trends in the development of conducting polymers-based biosensors	2019	TrAC—Trends in Analytical Chemistry	18	[88]

**Table 2:** Summary of the book chapters on poly(carbazole)derivatives extracted from Scopus.

Title	Author	Year	Book	Ref.
Light-emitting polymers	Perepichka, D.F., Perepichka, I.F., Meng, H., Wudl, F.	2006	Organic Light-Emitting Materials and Devices	[89]
Synthesis of Poly(2,7-carbazole)s and Derivatives	Boudreault, P.-L.T., Morin, J.-F., Leclerc, M.	2010	Design and Synthesis of Conjugated Polymers	[71]
Conducting polymer-based thermoelectric composites: Principles, processing, and applications	Yemata, T.A., Ye, Q., Zhou, H., (...), Chin, W.S., Xu, J.	2017	Hybrid Polymer Composite Materials: Applications	[90]
Light-emitting polymers	Xun, S., Perepichka, D.F., Perepichka, I.F., Meng, H., Wudl, F.	2017	Organic Light-Emitting Materials and Devices, Second Edition	[91]
Miscellaneous Vinyl Thermoplastics	Gilbert, M.	2017	Brydson’s Plastics Materials: Eighth Edition	[92]

## References

[1] Chiang C.K., Fincher C.R., Park Y.W., Heeger A.J., Shirakawa H., Louis E.J., Gau S.C., MacDiarmid A.G. (1977). Electrical conductivity in doped polyacetylene. Phys. Rev. Lett..

[2] Shirakawa H., Louis E.J., MacDiarmid A.G., Chiang C.K., Heeger A.J. (1977). Synthesis of electrically conducting organic polymers: Halogen derivatives of polyacetylene(CH)_x_. J. Chem. Soc. Chem. Commun..

[3] Zampetti A., Minotto A., Cacialli F. (2019). Near-Infrared (NIR) Organic Light-Emitting Diodes (OLEDs): Challenges and Opportunities. Adv. Funct. Mater..

[4] Izumi S., Higginbotham H.F., Nyga A., Stachelek P., Tohnai N., de Silva P., Data P., Takeda Y., Minakata S. (2020). Thermally Activated Delayed Fluorescent Donor–Acceptor–Donor–Acceptor π-Conjugated Macrocycle for Organic Light-Emitting Diodes. J. Am. Chem. Soc..

[5] Kim H.J., Lee C., Godumala M., Choi S., Park S.Y., Cho M.J., Park S., Choi D.H. (2018). Solution-processed thermally activated delayed fluorescence organic light-emitting diodes using a new polymeric emitter containing non-conjugated cyclohexane units. Polym. Chem..

[6] AlSalhi M.S., Alam J., Dass L.A., Raja M. (2011). Recent advances in conjugated polymers for light emitting devices. Int. J. Mol. Sci..

[7] Jiang Y., Guo Y., Liu Y. (2017). Engineering of Amorphous Polymeric Insulators for Organic Field-Effect Transistors. Adv. Electron. Mater..

[8] Kim D.E., Baeg K.-J. (2019). Reduction Treatment of Molecular-Doped Polymer Semiconductors for High-Performance N-Channel Organic Field-Effect Transistors. J. Korean Phys. Soc..

[9] Thuau D., Begley K., Dilmurat R., Ablat A., Wantz G., Ayela C., Abbas M. (2020). Exploring the Critical Thickness of Organic Semiconductor Layer for Enhanced Piezoresistive Sensitivity in Field-Effect Transistor Sensors. Materials.

[10] Baeg K.-J., Khim D., Kim J., Kim D.-Y., Sung S.-W., Yang B.-D., Noh Y.-Y. (2012). Flexible complementary logic gates using inkjet-printed polymer field-effect transistors. IEEE Electron Device Lett..

[11] Golmar F., Gobbi M., Llopis R., Stoliar P., Casanova F., Hueso L.E. (2012). Non-conventional metallic electrodes for organic field-effect transistors. Org. Electron..

[12] Keshtov M.L., Kuklin S.A., Konstantinov I.O., Khokhlov A.R., Dou C., Sharma G.D. (2020). Synthesis and Characterization of Wide-Bandgap Conjugated Polymers Consisting of Same Electron Donor and Different Electron-Deficient Units and Their Application for Nonfullerene Polymer Solar Cells. Macromol. Chem. Phys..

[13] Bildirir H., Di Carlo Rasi D., Wienk M.M., Janssen R.A.J., Avgeropoulos A., Gregoriou V.G., Allard S., Scherf U., Chochos C.L. (2018). New n-Type Solution Processable All Conjugated Polymer Network: Synthesis, Optoelectronic Characterization, and Application in Organic Solar Cells. Macromol. Rapid Commun..

[14] Scharber M.C. (2016). On the efficiency limit of conjugated polymer: Fullerene-based bulk heterojunction solar cells. Adv. Mater..

[15] Jung E.H., Ahn H., Jo W.H., Jo J.W., Jung J.W. (2019). Isoindigo-based conjugated polymer for high-performance organic solar cell with a high VOC of 1.06 V as processed from non-halogenated solvent. Dye. Pigment..

[16] Wong Y.-T., Lin P.-C., Tseng C.-W., Huang Y.-W., Su Y.-A., Chen W.-C., Chueh C.-C. (2020). Biaxially-extended side-chain engineering of benzodithiophene-based conjugated polymers and their applications in polymer solar cells. Org. Electron..

[17] Facchetti A. (2011). π-Conjugated polymers for organic electronics and photovoltaic cell applications. Chem. Mater..

[18] Sun L., Xu X., Song S., Zhang Y., Miao C., Liu X., Xing G., Zhang S. (2019). Medium-Bandgap Conjugated Polymer Donors for Organic Photovoltaics. Macromol. Rapid Commun..

[19] An C., Zheng Z., Hou J. (2020). Recent progress in wide bandgap conjugated polymer donors for high-performance nonfullerene organic photovoltaics. Chem. Commun..

[20] Brebels J., Manca J.V., Lutsen L., Vanderzande D., Maes W. (2017). High dielectric constant conjugated materials for organic photovoltaics. J. Mater. Chem. A.

[21] He Y., Hong W., Li Y. (2014). New building blocks for π-conjugated polymer semiconductors for organic thin film transistors and photovoltaics. J. Mater. Chem. C.

[22] Jia P., Hu T., He Q., Cao X., Ma J., Fan J., Chen Q., Ding Y., Pyun J., Geng J. (2018). Synthesis of a Macroporous Conjugated Polymer Framework: Iron Doping for Highly Stable, Highly Efficient Lithium–Sulfur Batteries. ACS Appl. Mater. Interfaces.

[23] Xu S., Wang G., Biswal B.P., Addicoat M., Paasch S., Sheng W., Zhuang X., Brunner E., Heine T., Berger R. (2019). A Nitrogen-Rich 2D sp 2 -Carbon-Linked Conjugated Polymer Framework as a High-Performance Cathode for Lithium-Ion Batteries. Angew. Chem. Int. Ed..

[24] Xie J., Gu P., Zhang Q. (2017). Nanostructured conjugated polymers: Toward high-performance organic electrodes for rechargeable batteries. ACS Energy Lett..

[25] Ma W., Zhang C., Gao X., Shu C., Yan C., Wang F., Chen Y., Zeng J.H., Jiang J.-X. (2020). Structure evolution of azo-fused conjugated microporous polymers for high performance lithium-ion batteries anodes. J. Power Sources.

[26] Gracia R., Mecerreyes D. (2013). Polymers with redox properties: Materials for batteries, biosensors and more. Polym. Chem..

[27] Wu Z., Chen L., Liu J., Parvez K., Liang H., Shu J., Sachdev H., Graf R., Feng X., Müllen K. (2014). High-performance electrocatalysts for oxygen reduction derived from cobalt porphyrin-based conjugated mesoporous polymers. Adv. Mater..

[28] Pandey R.K., Lakshminarayanan V. (2012). Ethanol electrocatalysis on gold and conducting polymer nanocomposites: A study of the kinetic parameters. Appl. Catal. B Environ..

[29] Ramanavičius A., Ramanavičienė A., Malinauskas A. (2006). Electrochemical sensors based on conducting polymer—Polypyrrole. Electrochim. Acta.

[30] Luo J.-H.H., Li Q., Chen S.-H.H., Yuan R. (2019). Coreactant-Free Dual Amplified Electrochemiluminescent Biosensor Based on Conjugated Polymer Dots for the Ultrasensitive Detection of MicroRNA. ACS Appl. Mater. Interfaces.

[31] Mercante L.A., Scagion V.P., Migliorini F.L., Mattoso L.H.C., Correa D.S. (2017). Electrospinning-based (bio) sensors for food and agricultural applications: A review. TrAC Trends Anal. Chem..

[32] Ates M. (2013). A review study of (bio)sensor systems based on conducting polymers. Mater. Sci. Eng. C.

[33] McCullough R.D. (1998). The chemistry of conducting polythiophenes. Adv. Mater..

[34] Leclerc M. (1999). Optical and electrochemical transducers based on functionalized conjugated polymers. Adv. Mater..

[35] Watanabe A., Murakami S., Mori K., Kashiwaba Y. (1989). Electronic properties of polypyrrole/n-Si heterojunctions and polypyrrole/metal contacts. Macromolecules.

[36] Rehahn M., Schlüter A.-D., Wegner G., Feast W.J. (1989). Soluble poly (para-phenylene) s. 1. Extension of the Yamamoto synthesis to dibromobenzenes substituted with flexible side chains. Polymer.

[37] Burroughes J.H., Bradley D.D.C., Brown A.R., Marks R.N., Mackay K., Friend R.H., Burns P.L., Holmes A.B. (1990). Light-emitting diodes based on conjugated polymers. Nature.

[38] Leclerc M. (2001). Polyfluorenes: Twenty years of progress. J. Polym. Sci. Part A Polym. Chem..

[39] Yang Y., Pei Q., Heeger A.J. (1996). Efficient blue light-emitting diodes from a soluble poly (para-phenylene) internal field emission measurement of the energy gap in semiconducting polymers. Synth. Met..

[40] Ranger M., Rondeau D., Leclerc M. (1997). New well-defined poly (2, 7-fluorene) derivatives: Photoluminescence and base doping. Macromolecules.

[41] Neher D. (2001). Polyfluorene homopolymers: Conjugated liquid-crystalline polymers for bright blue emission and polarized electroluminescence. Macromol. Rapid Commun..

[42] Collin G., Höke H., Talbiersky J. (2006). Carbazole. Ullmann’s Encyclopedia of Industrial Chemistry.

[43] Bashir M., Bano A., Ijaz A.S., Chaudhary B.A. (2015). Recent developments and biological activities of n-substituted carbazole derivatives: A review. Molecules.

[44] Nandy B.C., Gupta A.K., Mittal A., Vyas V. (2014). Carbazole: It’s biological activity. J. Biomed. Pharm. Res..

[45] Yavuz Ö., Sezer E., Saraç A.S. (2001). Spectroelectrochemical study of N-ethyl-carbazole in the presence of acrylamide. Polym. Int..

[46] Chen C.-H., Wang Y., Michinobu T., Chang S.-W., Chiu Y.-C., Ke C.-Y., Liou G.-S. (2020). Donor–Acceptor Effect of Carbazole-Based Conjugated Polymer Electrets on Photoresponsive Flash Organic Field-Effect Transistor Memories. ACS Appl. Mater. Interfaces.

[47] Rice N.A., Bodnaryk W.J., Mirka B., Melville O.A., Adronov A., Lessard B.H. (2019). Polycarbazole-Sorted Semiconducting Single-Walled Carbon Nanotubes for Incorporation into Organic Thin Film Transistors. Adv. Electron. Mater..

[48] Soganci T., Baygu Y., Gök Y., Ak M. (2018). Disulfide-linked symmetric N-alkyl carbazole derivative as a new electroactive monomer for electrochromic applications. Synth. Met..

[49] Ates M., Özyilmaz A.T., Özyılmaz A.T. (2015). The application of polycarbazole, polycarbazole/nanoclay and polycarbazole/Zn-nanoparticles as a corrosion inhibition for SS304 in saltwater. Prog. Org. Coat..

[50] Sun D., Ren Z., Bryce M.R., Yan S. (2015). Arylsilanes and siloxanes as optoelectronic materials for organic light-emitting diodes (OLEDs). J. Mater. Chem. C.

[51] Srivastava A., Chakrabarti P. (2017). Experimental characterization of electrochemically polymerized polycarbazole film and study of its behavior with different metals contacts. Appl. Phys. A.

[52] Wu C.-J.J., Gaharwar A.K., Schexnailder P.J., Schmidt G. (2010). Development of biomedical polymer-silicate nanocomposites: A materials science perspective. Materials.

[53] Pernites R., Ponnapati R., Felipe M.J., Advincula R. (2011). Electropolymerization molecularly imprinted polymer (E-MIP) SPR sensing of drug molecules: Pre-polymerization complexed terthiophene and carbazole electroactive monomers. Biosens. Bioelectron..

[54] Li J., Grimsdale A.C. (2010). Carbazole-based polymers for organic photovoltaic devices. Chem. Soc. Rev..

[55] Ci Z., Yu X., Bao M., Wang C., Ma T. (2013). Influence of the benzo [d] thiazole-derived π-bridges on the optical and photovoltaic performance of D–π–A dyes. Dye. Pigment..

[56] El-Emary T.I., El-Aal H.A.K.A., Mohamed S.K. (2018). Synthesis and Characterization of Assorted Heterocycles Based 3-(9Hcarbazol-9-yl) Propane Hydrazide. Chem. Sin..

[57] Zaia E.W., Gordon M.P., Yuan P., Urban J.J. (2019). Progress and Perspective: Soft Thermoelectric Materials for Wearable and Internet-of-Things Applications. Adv. Electron. Mater..

[58] Nitani M., Nakayama K., Maeda K., Omori M., Uno M. (2019). Organic temperature sensors based on conductive polymers patterned by a selective-wetting method. Org. Electron..

[59] Zhang Y., Park S.-J. (2019). Flexible organic thermoelectric materials and devices for wearable green energy harvesting. Polymers.

[60] Gao M., Li L., Song Y. (2017). Inkjet printing wearable electronic devices. J. Mater. Chem. C.

[61] Jeerapan I., Poorahong S. (2020). Review—Flexible and Stretchable Electrochemical Sensing Systems: Materials, Energy Sources, and Integrations. J. Electrochem. Soc..

[62] Amjadi M., Kyung K., Park I., Sitti M. (2016). Stretchable, skin-mountable, and wearable strain sensors and their potential applications: A review. Adv. Funct. Mater..

[63] Zeng W., Shu L., Li Q., Chen S., Wang F., Tao X. (2014). Fiber-based wearable electronics: A review of materials, fabrication, devices, and applications. Adv. Mater..

[64] Di J., Zhang X., Yong Z., Zhang Y., Li D., Li R., Li Q. (2016). Carbon-nanotube fibers for wearable devices and smart textiles. Adv. Mater..

[65] Russ B., Glaudell A., Urban J.J., Chabinyc M.L., Segalman R.A. (2016). Organic thermoelectric materials for energy harvesting and temperature control. Nat. Rev. Mater..

[66] Khan Y., Ostfeld A.E., Lochner C.M., Pierre A., Arias A.C. (2016). Monitoring of vital signs with flexible and wearable medical devices. Adv. Mater..

[67] Dong L., Xu C., Li Y., Huang Z.-H., Kang F., Yang Q.-H., Zhao X. (2016). Flexible electrodes and supercapacitors for wearable energy storage: A review by category. J. Mater. Chem. A.

[68] Kim J., Kumar R., Bandodkar A.J., Wang J. (2017). Advanced materials for printed wearable electrochemical devices: A review. Adv. Electron. Mater..

[69] Heo J.S., Eom J., Kim Y., Park S.K. (2018). Recent progress of textile-based wearable electronics: A comprehensive review of materials, devices, and applications. Small.

[70] Boudreault P.-L.T., Beaupré S., Leclerc M. (2010). Polycarbazoles for plastic electronics. Polym. Chem..

[71] Boudreault P.-L.T., Blouin N., Leclerc M. (2008). Poly(2,7-carbazole)s and Related Polymers. Polyfluorenes.

[72] Blouin N., Michaud A., Gendron D., Wakim S., Blair E., Neagu-Plesu R., Belletête M., Durocher G., Tao Y., Leclerc M. (2008). Toward a Rational Design of Poly(2,7-Carbazole) Derivatives for Solar Cells. J. Am. Chem. Soc..

[73] Wakim S., Aïch B., Tao Y., Leclerc M. (2008). Charge Transport, Photovoltaic, and Thermoelectric Properties of Poly(2,7-Carbazole) and Poly(Indolo[3,2-b]Carbazole) Derivatives. Polym. Rev..

[74] Morin J.-F., Leclerc M., Adès D., Siove A. (2005). Polycarbazoles: 25 Years of Progress. Macromol. Rapid Commun..

[75] Houben J.L., Natucci B., Solaro R., Colella O., Chiellini E., Ledwith A. (1978). Optically active vinyl polymers containing fluorescent groups: 5. Fluorescence properties of poly(9-vinyl carbazole) and optically active polymers containing carbazole units. Polymer.

[76] Murphy S.M., Hamilton C.J., Davies M.L., Tighe B.J. (1992). Polymer membranes in clinical sensor applications. Biomaterials.

[77] Grazulevicius J.V.V., Strohriegl P., Pielichowski J., Pielichowski K. (2003). Carbazole-containing polymers: Synthesis, properties and applications. Prog. Polym. Sci..

[78] Ding J., Gao J., Cheng Y., Xie Z., Wang L., Ma D., Jing X., Wang F. (2006). Highly Efficient Green-Emitting Phosphorescent Iridium Dendrimers Based on Carbazole Dendrons. Adv. Funct. Mater..

[79] Faridbod F., Norouzi P., Dinarvand R., Ganjali M. (2008). Developments in the Field of Conducting and Non-conducting Polymer Based Potentiometric Membrane Sensors for Ions Over the Past Decade. Sensors.

[80] Zou Y., Gendron D., Badrou-Aïch R., Najari A., Tao Y., Leclerc M. (2009). A High-Mobility Low-Bandgap Poly(2,7-carbazole) Derivative for Photovoltaic Applications. Macromolecules.

[81] Ates M., Sarac A.S. (2009). Conducting polymer coated carbon surfaces and biosensor applications. Prog. Org. Coat..

[82] Beaupré S., Boudreault P.-L.T., Leclerc M. (2010). Solar-Energy Production and Energy-Efficient Lighting: Photovoltaic Devices and White-Light-Emitting Diodes Using Poly(2,7-fluorene), Poly(2,7-carbazole), and Poly(2,7-dibenzosilole) Derivatives. Adv. Mater..

[83] Dubey N., Leclerc M. (2011). Conducting polymers: Efficient thermoelectric materials. J. Polym. Sci. Part B Polym. Phys..

[84] Gendron D., Leclerc M. (2011). New conjugated polymers for plastic solar cells. Energy Environ. Sci..

[85] Grigoras A.G. (2016). A review on medical applications of poly(N-vinylcarbazole) and its derivatives. Int. J. Polym. Mater. Polym. Biomater..

[86] Tan S.E., Sarjadi M.S. (2017). The recent development of carbazole-, benzothiadiazole-, and isoindigo-based copolymers for solar cells application: A review. Polym. Sci. Ser. B.

[87] Liguori R., Botta A., Rubino A., Pragliola S., Venditto V. (2018). Stereoregular polymers with pendant carbazolyl groups: Synthesis, properties and optoelectronic applications. Synth. Met..

[88] Ghorbani Zamani F., Moulahoum H., Ak M., Odaci Demirkol D., Timur S. (2019). Current trends in the development of conducting polymers-based biosensors. TrAC Trends Anal. Chem..

[89] Perepichka D.F., Perepichka I.F., Meng H., Wudl F. (2006). Light-emitting polymers. Organic Light-Emitting Materials and Devices.

[90] Yemata T.A., Ye Q., Zhou H., Kyaw A.K.K., Chin W.S., Xu J. (2017). Conducting polymer-based thermoelectric composites. Hybrid Polymer Composite Materials.

[91] Li Z.R. (2017). Organic Light-Emitting Materials and Devices.

[92] Gilbert M. (2017). Miscellaneous Vinyl Thermoplastics. Brydson’s Plastics Materials.

[93] Michinobu T., Osako H., Shigehara K. (2010). Synthesis and Properties of 1,8-Carbazole-Based Conjugated Copolymers. Polymers.

[94] Michinobu T., Osako H., Shigehara K. (2009). Synthesis and Properties of Conjugated Poly(1,8-carbazole)s. Macromolecules.

[95] Branch G.E.K., Smith J.F. (1920). A bivalent nitrogen derivative of carbazole. J. Am. Chem. Soc..

[96] Perkin W.H., Tucker S.H. (1921). XXVI.—The oxidation of carbazole. J. Chem. Soc. Trans..

[97] Branch G.E.K., Hall W.W. (1924). Oxidation of carbazole by silver oxide. J. Am. Chem. Soc..

[98] Aoai T., Nishio R., Hayashi N., Nomura K. (2016). Photo Doping Process of Conductive Polymer with PAG and Application for Organic Thermoelectric Materials. J. Photopolym. Sci. Technol..

[99] Sangwan W., Paradee N., Sirivat A. (2016). Polycarbazole by chemical oxidative interfacial polymerization: Morphology and electrical conductivity based on synthesis conditions. Polym. Int..

[100] Shakir M., Noor-e-Iram, Khan M.S., Al-Resayes S.I., Khan A.A., Baig U. (2014). Electrical Conductivity, Isothermal Stability, and Ammonia-Sensing Performance of Newly Synthesized and Characterized Organic–Inorganic Polycarbazole–Titanium Dioxide Nanocomposite. Ind. Eng. Chem. Res..

[101] Baig U., Wani W.A., Ting Hun L. (2015). Facile synthesis of an electrically conductive polycarbazole–zirconium(IV)phosphate cation exchange nanocomposite and its room temperature ammonia sensing performance. New J. Chem..

[102] Gupta B., Prakash R. (2010). Interfacial polymerization of carbazole: Morphology controlled synthesis. Synth. Met..

[103] Sun Q., Deng Y. (2005). In situ synthesis of temperature-sensitive hollow microspheres via interfacial polymerization. J. Am. Chem. Soc..

[104] Gupta B., Joshi L., Prakash R. (2011). Novel Synthesis of Polycarbazole-Gold Nanocomposite. Macromol. Chem. Phys..

[105] Gupta B., Prakash R., Melvin A. (2012). Chemical Synthesis of Polycarbazole (PCz), modification and pH sensor application. Proc. Int. Conf. Sens. Technol. ICST.

[106] Kumar A., Tiwari M., Prakash R. (2015). Electrochemical Study of Interfacially Synthesized Polycarbazole with Different Oxidants. ChemElectroChem.

[107] Balun Kayan D., Polat V. (2017). Improvement of electrochemical and structural properties of polycarbazole by simultaneous electrodeposition of chitosan. TURKISH J. Chem..

[108] Li C., Xue J., Huang A., Ma J., Qing F., Zhou A., Wang Z., Wang Y., Li J. (2019). Poly (N-vinylcarbazole) as an advanced organic cathode for potassium-ion-based dual-ion battery. Electrochim. Acta.

[109] Raies A. (2015). Elaboration de Films Minces Électroluminescents à Base de Polymère Conducteur Électronique et de Nanotubes de Carbone. Ph.D. Thesis.

[110] Zotti G., Schiavon G. (1984). Poly(2,5-thienylene)-coated electrodes formed by electroreduction of a nickel adduct with 2,5-dibromothiophene. J. Electroanal. Chem. Interfacial Electrochem..

[111] Schiavon G., Zotti G., Bontempelli G. (1984). An electroactive nickel containing polymeric film obtained by electrochemical reduction of an aryl-nickel derivative. J. Electroanal. Chem. Interfacial Electrochem..

[112] Xu Z., Horowitz G., Garnier F. (1988). Cathodic electropolymerization of polythiophene on platinum and various semiconducting electrodes. J. Electroanal. Chem. Interfacial Electrochem..

[113] Id H.A.L. (2014). Synthèse Par Voie Électrochimique de Nanostructures de Polymères Conducteurs Sans Emploi d ’ une Matrice Support: Applications Aux (bio) Capteurs. Ph.D. Thesis.

[114] Balint R., Cassidy N.J., Cartmell S.H. (2014). Conductive polymers: Towards a smart biomaterial for tissue engineering. Acta Biomater..

[115] Ambrose J.F., Nelson R.F. (1968). Anodic Oxidation Pathways of Carbazoles. J. Electrochem. Soc..

[116] Lévesque I., Bertrand P.O., Blouin N., Leclerc M., Zecchin S., Zotti G., Ratcliffe C.I., Klug D.D., Gao X., Gao F. (2007). Synthesis and thermoelectric properties of polycarbazole, polyindolocarbazole, and polydiindolocarbazole derivatives. Chem. Mater..

[117] Zotti G., Schiavon G., Zecchin S., Morin J.F., Leclerc M. (2002). Electrochemical, conductive, and magnetic properties of 2,7-carbazole-based conjugated polymers. Macromolecules.

[118] Ates M., Sarac A.S. (2009). Electrochemical impedance spectroscopy of poly[carbazole-co-N-p-tolylsulfonyl pyrrole] on carbon fiber microelectrodes, equivalent circuits for modelling. Prog. Org. Coat..

[119] Penwell R.C. (1978). Poly(N-vinylcarbazole): A Selective Review of its Polymerization, Structure, Properties, and electrical characteristics. J. Polym. Sci. Macromol. Rev..

[120] Tran-Van F., Henri T., Chevrot C. (2002). Synthesis and electrochemical properties of mixed ionic and electronic modified polycarbazole. Electrochim. Acta.

[121] Sotzing G.A., Reddinger J.L., Katritzky A.R., Soloducho J., Musgrave R., Reynolds J.R., Steel P.J. (1997). Multiply Colored Electrochromic Carbazole-Based Polymers. Chem. Mater..

[122] Reppe W., Keyssner E. (1935). N-Vinyl Compounds. DRP.

[123] Baethge H., Butz S., Schmidt-Naake G. (1997). “Living” free radical copolymerization of styrene and N-vinylcarbazole. Macromol. Rapid Commun..

[124] Kim W., Nishikawa Y., Watanabe H., Kanazawa A., Aoshima S., Fujii A., Ozaki M. (2019). Stereoregularity effect on hole mobility in poly (N-vinylcarbazole) thin film evaluated by MIS-CELIV method. Jpn. J. Appl. Phys..

[125] Natori I. (2006). Anionic Polymerization of N-Vinylcarbazole with Alkyllithium as an Initiator. Macromolecules.

[126] Wang T., Yang C., Shieh Y., Yeh A. (2009). Synthesis of CdSe–Poly (N-vinylcarbazole) Nanocomposite by Atom Transfer Radical Polymerization for Potential Optoelectronic Applications. Macromol. Rapid Commun..

[127] Nakabayashi K., Mori H. (2013). Recent progress in controlled radical polymerization of N-vinyl monomers. Eur. Polym. J..

[128] Hawker C.J., Bosman A.W., Harth E. (2001). New polymer synthesis by nitroxide mediated living radical polymerizations. Chem. Rev..

[129] Natsuume T., Akana Y., Tanabe K., Fujimatsu M., Shimizu M., Shirota Y., Hirata H., Kusabayashi S., Mikawa H. (1969). Mechanism of charge-transfer polymerizations: Polymerization of N-vinylcarbazole with tetrachloro-p-benzoquinone in benzene. J. Chem. Soc. D Chem. Commun..

[130] Hazra D.K., Chatterjee R. (2013). In situ solid state polymerization and characterization of poly (N-vinylcarbazole) encapsulated Keggin type polyoxometalate nanocomposite. J. Mol. Struct..

[131] Hurtgen M., Detrembleur C., Jerome C., Debuigne A. (2011). Insight into organometallic-mediated radical polymerization. Polym. Rev..

[132] Marimuthu E., Murugesan V. (2019). Influence of ultrasound on multi-site phase transfer catalyzed polymerization of N-vinyl carbazole in two phase system. SN Appl. Sci..

[133] Frau A.F., Pernites R.B., Advincula R.C. (2010). A Conjugated Polymer Network Approach to Anticorrosion Coatings: Poly(vinylcarbazole) Electrodeposition. Ind. Eng. Chem. Res..

[134] Huang W., Gu C.C., Wang T., Gu C.C., Qiao S., Yang R. (2014). Effect of two facile synthetic strategies with alterable polymerization sequence on the performance of N-vinyl carbazole-based conjugated porous materials. RSC Adv..

[135] Liu G., Chen Y., Li R.W., Zhang B., Kang E.T., Wang C., Zhuang X. (2014). Resistance-Switchable Graphene Oxide-Polymer Nanocomposites for Molecular Electronics. ChemElectroChem.

[136] Santos C.M., Tria M.C.R., Vergara R.A.M.V., Cui K.M., Pernites R., Advincula R.C. (2011). Films of Highly Disperse Electrodeposited Poly(N-vinylcarbazole)–Graphene Oxide Nanocomposites. Macromol. Chem. Phys..

[137] Wang C., Xin G., Lee Y., Hao J., Jiang J., Liu H. (2010). Poly (9-vinylcarbazole)/silver composite nanotubes and networks formed at the air–water interface. J. Appl. Polym. Sci..

[138] Aydın A., Kaya İ. (2013). Syntheses of novel copolymers containing carbazole and their electrochromic properties. J. Electroanal. Chem..

[139] Aydın A., Kaya İ.I. (2012). Synthesis and characterization of yellow and green light emitting novel polymers containing carbazole and electroactive moieties. Electrochim. Acta.

[140] Kocaeren A.A. (2016). Synthesis and characterization of novel polymers based on carbazole with NaOCl and FeCl3 oxidants. Iran. Polym. J..

[141] Hsiao S.H., Lin S.W. (2016). Electrochemical synthesis of electrochromic polycarbazole films from N-phenyl-3,6-bis(N-carbazolyl)carbazoles. Polym. Chem..

[142] Kocaeren A.A. (2015). Electrochemical synthesis and electrochromic application of a novel polymer based on carbazole. Org. Electron..

[143] Hsiao S.-H., Hsueh J.-C. (2015). Electrochemical synthesis and electrochromic properties of new conjugated polycarbazoles from di(carbazol-9-yl)-substituted triphenylamine and N-phenylcarbazole derivatives. J. Electroanal. Chem..

[144] Yang X.-L.L., Hu D.-Y.Y., Chen Q., Li L., Li P.-X.X., Ren S.-B.B., Bertuzzo M., Chen K., Han D.-M.M., Zhou X.-H.H. (2019). A pyrene-cored conjugated microporous polycarbazole for sensitive and selective detection of hazardous explosives. Inorg. Chem. Commun..

[145] Duran B., Çakmakcı Ünver İ., Bereket G., Ünver İ.Ç., Bereket G. (2017). Inhibition of steel corrosion by potentiodynamic deposition of poly(N-methyl carbazole). J. Adhes. Sci. Technol..

[146] Elkhidr H.E., Ertekin Z., Udum Y.A., Pekmez K. (2020). Electrosynthesis and characterizations of electrochromic and soluble polymer films based on N-substituted carbazole derivates. Synth. Met..

[147] Qin L., Zhang S., Xu J., Lu B., Duan X., Zhu D., Huang Y. (2013). Novel poly(ethylene oxide) grafted polycarbazole conjugated freestanding network films via anionic and electrochemical polymerization. Int. J. Electrochem. Sci..

[148] Li W., Otsuka M., Kato T., Wang Y., Mori T., Michinobu T. (2016). 3,6-Carbazole vs 2,7-carbazole: A comparative study of hole-transporting polymeric materials for inorganic-organic hybrid perovskite solar cells. Beilstein J. Org. Chem..

[149] Tao X.-T., Zhang Y.-D., Wada T., Sasabe H., Suzuki H., Watanabe T., Miyata S. (1998). Hyperbranched Polymers for Electroluminescence Applications. Adv. Mater..

[150] Aïch R.B., Blouin N., Bouchard A., Leclerc M. (2009). Electrical and Thermoelectric Properties of Poly(2,7-Carbazole) Derivatives. Chem. Mater..

[151] Morin J.-F., Leclerc M. (2001). Syntheses of Conjugated Polymers Derived from N -Alkyl-2,7-carbazoles. Macromolecules.

[152] Nguyen Q., Baek S., Kim M., Shin N., Kim G., Choe D., Kwon J., Chai K. (2014). Novel Hole Transporting Materials Based on 4-(9H-Carbazol-9-yl)triphenylamine Derivatives for OLEDs. Molecules.

[153] Zou S.-J., Shen Y., Xie F.-M., Chen J.-D., Li Y.-Q., Tang J.-X. (2020). Recent advances in organic light-emitting diodes: Toward smart lighting and displays. Mater. Chem. Front..

[154] Hebner T.R., Wu C.C., Marcy D., Lu M.H., Sturm J.C. (1998). Ink-jet printing of doped polymers for organic light emitting devices. Appl. Phys. Lett..

[155] Krucaite G., Grigalevicius S., Gupta B., Joshi L., Prakash R. (2019). A review on low-molar-mass carbazole- based derivatives for organic light emitting diodes. Synth. Met..

[156] Bhuvana K.P., Joseph Bensingh R., Abdul Kader M., Nayak S.K. (2018). Polymer light emitting diodes: Materials, technology and device. Polym. Plast. Technol. Eng..

[157] Dumur F. (2015). Carbazole-based polymers as hosts for solution-processed organic light-emitting diodes: Simplicity, efficacy. Org. Electron..

[158] Syutkin R.V., Abashev G.G., Shklyaeva E.V., Kudryavtsev P.G. (2011). New carbazole-containing chalcones and pyrimidines based thereon: Synthesis and electrochemical study. Russ. J. Org. Chem..

[159] Wang H., Lin J., Shen Z.X. (2016). Polyaniline (PANi) based electrode materials for energy storage and conversion. J. Sci. Adv. Mater. Devices.

[160] Li J., Cheng X., Shashurin A., Keidar M. (2012). Review of Electrochemical Capacitors Based on Carbon Nanotubes and Graphene. Graphene.

[161] Chen Q., Shen Y., Zhang S., Zhang Q.M. (2015). Polymer-Based Dielectrics with High Energy Storage Density. Annu. Rev. Mater. Res..

[162] An K.H.H., Jeon K.K.K., Heo J.K.K., Lim S.C.C., Bae D.J.J., Lee Y.H.H. (2002). High-Capacitance Supercapacitor Using a Nanocomposite Electrode of Single-Walled Carbon Nanotube and Polypyrrole. J. Electrochem. Soc..

[163] Rudge A., Raistrick I., Gottesfeld S., Ferraris J.P. (1994). A study of the electrochemical properties of conducting polymers for application in electrochemical capacitors. Electrochim. Acta.

[164] Ryu K.S., Kim K.M., Park N.-G., Park Y.J., Chang S.H. (2002). Symmetric redox supercapacitor with conducting polyaniline electrodes. J. Power Sources.

[165] Ates M. (2016). Graphene and its nanocomposites used as an active materials for supercapacitors. J. Solid State Electrochem..

[166] Yiğit D., Güllü M. (2018). Capacitive properties of novel N-alkyl substituted poly(3,6-dithienyl-9H-carbazole)s as redox electrode materials and their symmetric micro-supercapacitor applications. Electrochim. Acta.

[167] Snook G.A., Kao P., Best A.S. (2011). Conducting-polymer-based supercapacitor devices and electrodes. J. Power Sources.

[168] Ates M., Uludag N. (2015). Poly(9H-Carbazole-9-Carbothioic Dithioperoxyanhydride) Formation and Capacitor Study. Int. J. Polym. Mater. Polym. Biomater..

[169] Wang H., Cheng Z., Liao Y., Li J., Weber J., Thomas A., Faul C.F.J. (2017). Conjugated Microporous Polycarbazole Networks as Precursors for Nitrogen-Enriched Microporous Carbons for CO 2 Storage and Electrochemical Capacitors. Chem. Mater..

[170] Duran B., Ünver İ.Ç., Bereket G. (2020). Investigation of Supporting Electrolyte Effect on Supercapacitor Properties of Poly (Carbazole) Films. J. Electrochem. Sci. Technol..

[171] Zhang B., Li B., Wang Z. (2020). Creation of Carbazole-Based Fluorescent Porous Polymers for Recognition and Detection of Various Pesticides in Water. ACS Sens..

[172] Cheng Z., Dai Z., Li J., Wang H., Huang M.-R.R., Li X.-G.G., Liao Y. (2019). Template-free synthesis of tunable hollow microspheres of aniline and aminocarbazole copolymers emitting colorful fluorescence for ultrasensitive sensors. Chem. Eng. J..

[173] Sathiyan G., Sivakumar E.K.T.K.T., Ganesamoorthy R., Thangamuthu R., Sakthivel P. (2016). Review of carbazole based conjugated molecules for highly efficient organic solar cell application. Tetrahedron Lett..

[174] Zafer C., Gultekin B., Ozsoy C., Tozlu C., Aydin B., Icli S. (2010). Carbazole-based organic dye sensitizers for efficient molecular photovoltaics. Sol. Energy Mater. Sol. Cells.

[175] Akhundi A., Habibi-Yangjeh A., Abitorabi M., Rahim Pouran S. (2019). Review on photocatalytic conversion of carbon dioxide to value-added compounds and renewable fuels by graphitic carbon nitride-based photocatalysts. Catal. Rev..

[176] Zhang Z., Liao M., Lou H., Hu Y., Sun X., Peng H. (2018). Conjugated Polymers for Flexible Energy Harvesting and Storage. Adv. Mater..

[177] Islam G.M.N., Ali A., Collie S. (2020). Textile sensors for wearable applications: A comprehensive review. Cellulose.

[178] Fujita H., Michinobu T. (2012). Synthesis and photovoltaic properties of 1,8-carbazole-based donor-acceptor type conjugated polymers. Macromol. Chem. Phys..

[179] Qin R., Yang J., Li P., Wu Q., Zhou Y., Luo H., Chang F. (2016). Structure property relationship for carbazole and benzothiadiazole based conjugated polymers. Sol. Energy Mater. Sol. Cells.

[180] Yen H.-J.J., Shan C., Wang L., Xu P., Zhou M., Wang H.-L.L. (2017). Development of conjugated polymers for memory device applications. Polymers.

[181] Liu C.-L., Chen W.-C. (2011). Donor–acceptor polymers for advanced memory device applications. Polym. Chem..

[182] Wu H.-C., Yu A.-D., Lee W.-Y., Liu C.-L., Chen W.-C. (2012). A poly (fluorene-thiophene) donor with a tethered phenanthro [9, 10-d] imidazole acceptor for flexible nonvolatile flash resistive memory devices. Chem. Commun..

[183] Elsawy W., Son M., Jang J., Kim M.J., Ji Y., Kim T.W., Ko H.C., Elbarbary A., Ham M.H., Lee J.S. (2015). Isoindigo-Based Donor-Acceptor Conjugated Polymers for Air-Stable Nonvolatile Memory Devices. ACS Macro Lett..

[184] Zha D., Chen L., Wu F., Wang H., Chen Y. (2013). Donor-acceptor-integrated conjugated polymers based on carbazole[3,4-c:5,6- c]bis[1,2,5]thiadiazole with tight π-π Stacking for photovoltaics. J. Polym. Sci. Part A Polym. Chem..

[185] Chem P., Liu C., Chen W. (2011). Polymer Chemistry MINIREVIEW Donor—Acceptor polymers for advanced memory device applications. Polym. Chem..

[186] Lin W.P., Liu S.J., Gong T., Zhao Q., Huang W. (2014). Polymer-based resistive memory materials and devices. Adv. Mater..

[187] Hahm S.G., Lee T.J., Kim D.M., Kwon W., Ko Y.G., Michinobu T., Ree M. (2011). Electrical memory characteristics of nitrogen-linked poly(2,7-carbazole)s. J. Phys. Chem. C.

[188] Zhang B., Liu G., Chen Y., Wang C., Neoh K.G., Bai T., Kang E.T. (2012). Electrical bistability and WORM memory effects in donor-acceptor polymers based on poly(N-vinylcarbazole). Chempluschem.

[189] Kang N., Cho B., Kang B., Song S., Lee T., Lee J. (2012). Structural and Electrical Characterization of a Block Copolymer-Based Unipolar Nonvolatile Memory Device. Adv. Mater..

